# Supplementary material for: Protection-Free Strategy for the Synthesis of Boro-Depsipeptides in Aqueous Media under Microwave-Assisted Conditions
Source: Molecules. 2022 Apr 4;27(7):2325. doi: 10.3390/molecules27072325 (PMC9000886; doi:10.3390/molecules27072325)
Supplement: Supplementary file 1 [file molecules-27-02325-s001.zip › molecules-1595292-supplementary.pdf]

Supplementary Materials

Protection-free Strategy for the Synthesis of Boro-depsipeptides in Aqueous Media under Microwave-assisted Conditions

Shuo-Bei Qiu [1], Pei-Yao Liu [2], Bo-Cheng Wang [3], Pin-Rui Chen [3], Jing-Han Xiao [3], Ting-Yu Hsu [3], Kuan-Lin Pan [3], Zhi-Yin Lai [3], Yi-Wei Chen [4], Ying-Chuan Chen [2,*], Jen-Kun Chen [1,5,*] and Po-Shen Pan [3,*]

1 Graduate Institute of Life Sciences, National Defense Medical Center, No.161, Sec. 6, Minquan E. Rd., Neihu Dist., Taipei City 11490, Taiwan, Republic of China; gloria919147@gmail.com
2 Department of Physiology & Biophysics, National Defense Medical Center, No.161, Sec. 6, Minquan E. Rd., Neihu Dist., Taipei City 11490, Taiwan, Republic of China; peiyaol01@gmail.com
3 Department of Chemistry, College of Sciences, Tamkang University, No. 151, Yingzhuan Rd., Danshui Dist., New Taipei City 25137, Taiwan, Republic of China; bcw@mail.tku.edu.tw (B.-C.W.); shawn19991002@gmail.com (P.-R.C.); a0952389829@gmail.com (J.-H.X.); lydia1020552@gmail.com (T.-Y.H.); ss870119@gmail.com (K.-L.P.); aa0935900324@gmail.com (Z.-Y.L.)
4 Department of Oncology, Taipei Veterans General Hospital. No. 201, Sec. 2, Shipai Rd., Beitou District, Taipei City, Taiwan 11217, Republic of China; chenyw@vghtpe.gov.tw
5 Center for Nanomedicine Research, National Health Research Institutes, Zhunan, Miaoli County 35053, Taiwan, Republic of China
* Correspondence: addy0918@gmail.com (Y.-C.C.); jkchen@nhri.edu.tw (J.-K.C.); 138020@gms.tku.edu.tw (P.-S.P.)

Table of Content

Figure S1. The ^{1}H NMR spectrum of **4a** in CD_3OD

Figure S2. The ^{13}C NMR spectrum of **4a** in CD_3OD

Figure S3. The ^{11}B NMR spectrum of **4a** in CD_3OD

Figure S4. The HRMS (ESI^+) of spectrum of **4a**

Figure S5. The ^{1}H NMR spectrum of **4b** in CD_3OD

Figure S6. The ^{13}C NMR spectrum of **4b** in CD_3OD

Figure S7. The ^{11}B NMR spectrum of **4b** in CD_3OD

Figure S8. The HRMS (ESI^+) of spectrum of **4b**

Figure S9. The ^{1}H NMR spectrum of **4c** in CD_3OD

Figure S10. The ^{13}C NMR spectrum of **4c** in CD_3OD

Figure S11. The ^{11}B NMR spectrum of **4c** in CD_3OD

Figure S12. The HRMS (ESI^+) of spectrum of **4c**

Figure S13. The ^{1}H NMR spectrum of **4d** in CD_3OD

Figure S14. The ^{13}C NMR spectrum of **4d** in CD_3OD

Figure S15. The ^{11}B NMR spectrum of **4d** in CD_3OD

Figure S16. The HRMS (ESI^+) of spectrum of **4d**

Figure S17. The ^{1}H NMR spectrum of **4e** in CD_3OD

Figure S18. The ^{13}C NMR spectrum of **4e** in CD_3OD

Figure S19. The ^{11}B NMR spectrum of **4e** in CD_3OD
Figure S20. The HRMS (ESI^+) of spectrum of **4e**
Figure S21. The ^{1}H NMR spectrum of **4f** in CD_3OD
Figure S22. The ^{13}C NMR spectrum of **4f** in CD_3OD
Figure S23. The ^{11}B NMR spectrum of **4f** in CD_3OD
Figure S24. The HRMS (ESI^+) of spectrum of **4f**
Figure S25. The ^{1}H NMR spectrum of **4g** in CD_3OD
Figure S26. The ^{13}C NMR spectrum of **4g** in CD_3OD
Figure S27. The ^{11}B NMR spectrum of **4g** in CD_3OD
Figure S28. The HRMS (ESI^+) of spectrum of **4g**
Figure S29. The ^{1}H NMR spectrum of **5a** in CD_3OD
Figure S30. The ^{13}C NMR spectrum of **5a** in CD_3OD
Figure S31. The ^{11}B NMR spectrum of **5a** in CD_3OD
Figure S32. The HRMS (ESI^+) of spectrum of **5a**
Figure S33. The ^{1}H NMR spectrum of **5b** in CD_3OD
Figure S34. The ^{13}C NMR spectrum of **5b** in CD_3OD
Figure S35. The ^{11}B NMR spectrum of **5b** in CD_3OD
Figure S36. The HRMS (ESI^+) of spectrum of **5b**
Figure S37. The ^{1}H NMR spectrum of **5c** in CD_3OD
Figure S38. The ^{13}C NMR spectrum of **5c** in CD_3OD
Figure S39. The ^{11}B NMR spectrum of **5c** in CD_3OD
Figure S40. The HRMS (ESI^+) of spectrum of **5c**
Figure S41. The ^{1}H NMR spectrum of **5d** in CD_3OD
Figure S42. The ^{13}C NMR spectrum of **5d** in CD_3OD
Figure S43. The ^{11}B NMR spectrum of **5d** in CD_3OD
Figure S44. The HRMS (ESI^+) of spectrum of **5d**
Figure S45. The ^{1}H NMR spectrum of **5e** in CD_3OD
Figure S46. The ^{13}C NMR spectrum of **5e** in CD_3OD
Figure S47. The ^{11}B NMR spectrum of **5e** in CD_3OD
Figure S48. The HRMS (ESI^+) of spectrum of **5e**
Figure S49. The ^{1}H NMR spectrum of **5f** in CD_3OD
Figure S50. The ^{13}C NMR spectrum of **5f** in $CDCl_3$
Figure S51. The ^{11}B NMR spectrum of **5f** in CD_3OD
Figure S52. The HRMS (ESI^+) of spectrum of **5f**
Figure S53. The ^{1}H NMR spectrum of **5g** in CD_3OD
Figure S54. The ^{13}C NMR spectrum of **5g** in CD_3OD
Figure S55. The ^{11}B NMR spectrum of **5g** in CD_3OD
Figure S56. The HRMS (ESI^+) of spectrum of **5g**

Figure S57. The ^{1}H NMR spectrum of **5h** in CD_3OD
Figure S58. The ^{13}C NMR spectrum of **5h** in CD_3OD
Figure S59. The ^{11}B NMR spectrum of **5h** in CD_3OD
Figure S60. The HRMS (ESI^+) of spectrum of **5h**
Figure S61. The ^{1}H NMR spectrum of **5i** in CD_3OD
Figure S62. The ^{13}C NMR spectrum of **5i** in CD_3OD
Figure S63. The ^{11}B NMR spectrum of **5i** in CD_3OD
Figure S64. The HRMS (ESI^+) of spectrum of **5i**
Figure S65. The ^{1}H NMR spectrum of **5j** in CD_3OD
Figure S66. The ^{13}C NMR spectrum of **5j** in CD_3OD
Figure S67. The ^{11}B NMR spectrum of **5j** in CD_3OD
Figure S68. The HRMS (ESI^+) of spectrum of **5j**
Figure S69. The ^{1}H NMR spectrum of **5k** in CD_3OD
Figure S70. The ^{13}C NMR spectrum of **5k** in CD_3OD
Figure S71. The ^{11}B NMR spectrum of **5k** in CD_3OD
Figure S72. The HRMS (ESI^-) of spectrum of **5k**
Figure S73. The ^{1}H NMR spectrum of **5l** in CD_3OD
Figure S74. The ^{13}C NMR spectrum of **5l** in CD_3OD
Figure S75. The HRMS (ESI^+) of spectrum of **5l**
Figure S76. The cytotoxicity of the compounds **4a-4d**, **4f** and **4g** in HEK-293 cells.
Figure S77. The cytotoxicity of the compounds **5b-5g**, **5j** and **5k** in HEK-293 cells.

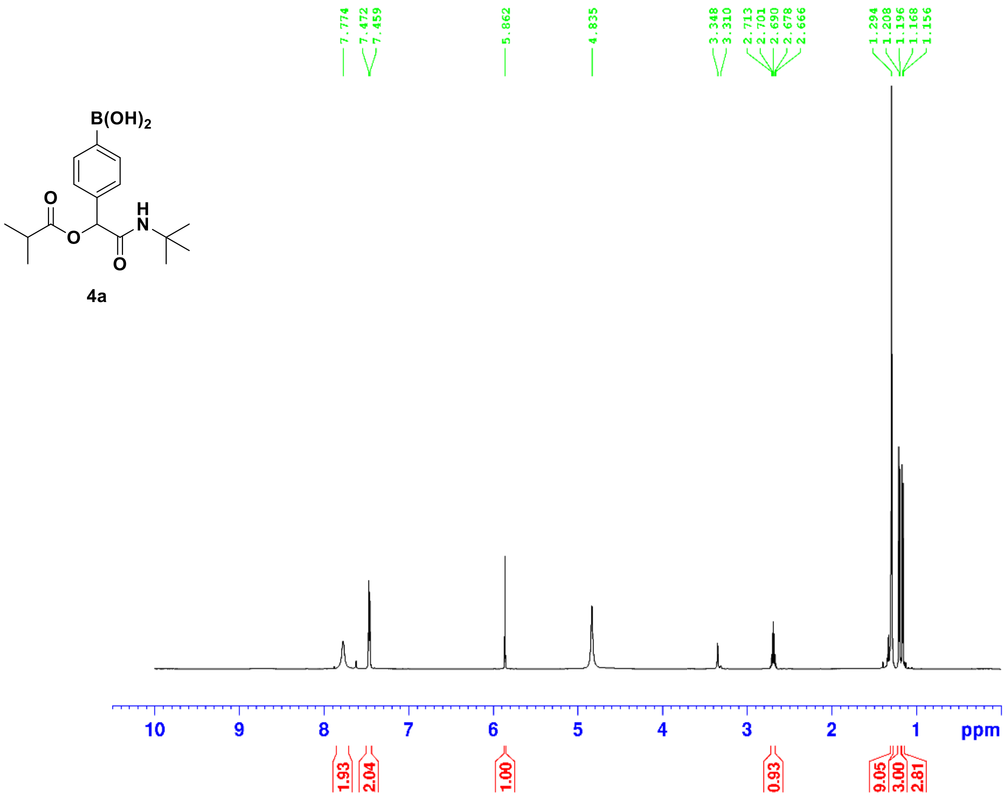

Figure S1. The ^{1}H NMR spectrum of **4a** in CD_3OD

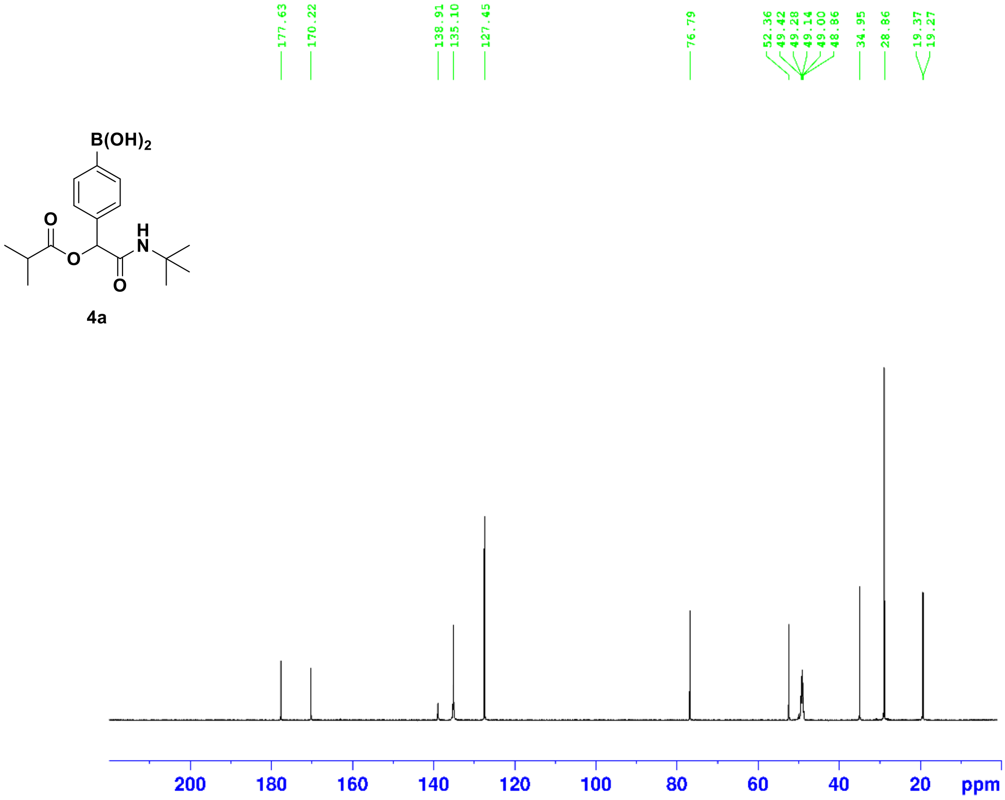

Figure S2. The ^{13}C NMR spectrum of **4a** in CD_3OD

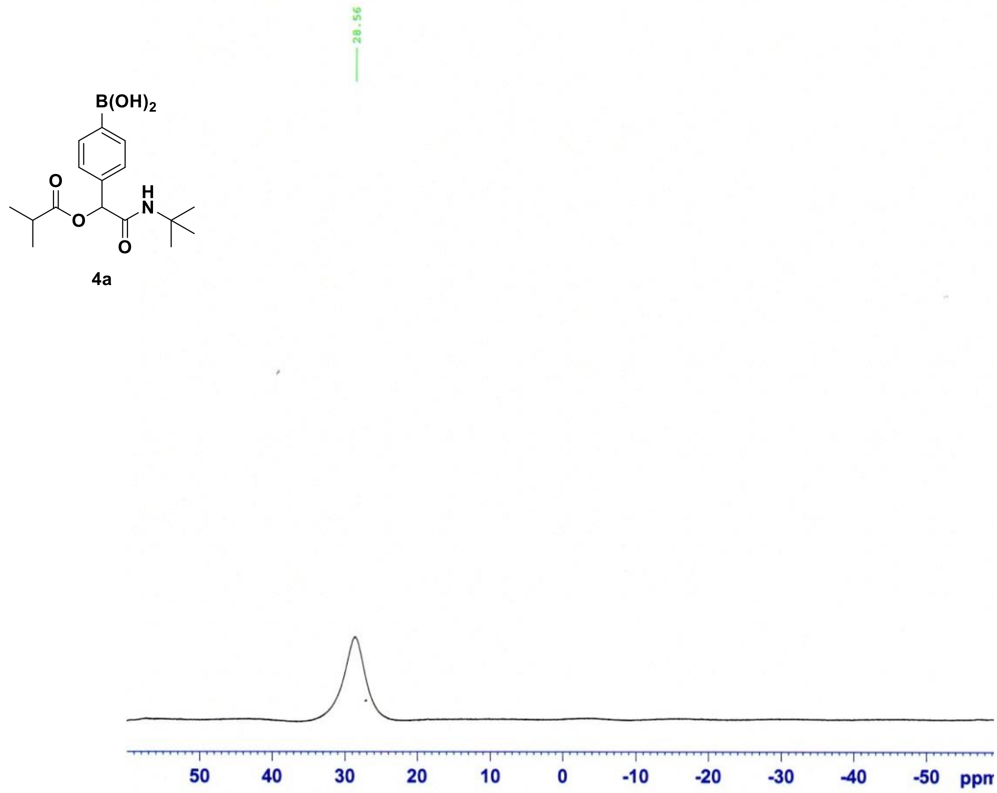

Figure S3. The ^{11}B NMR spectrum of **4a** in CD_3OD

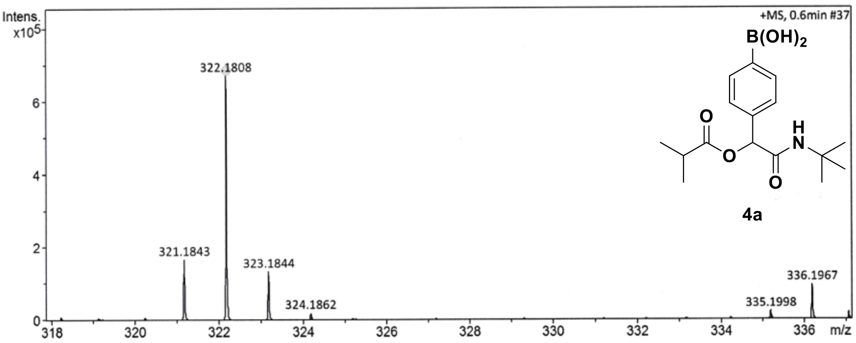

Figure S4. The HRMS (ESI$^+$) of spectrum of **4a**

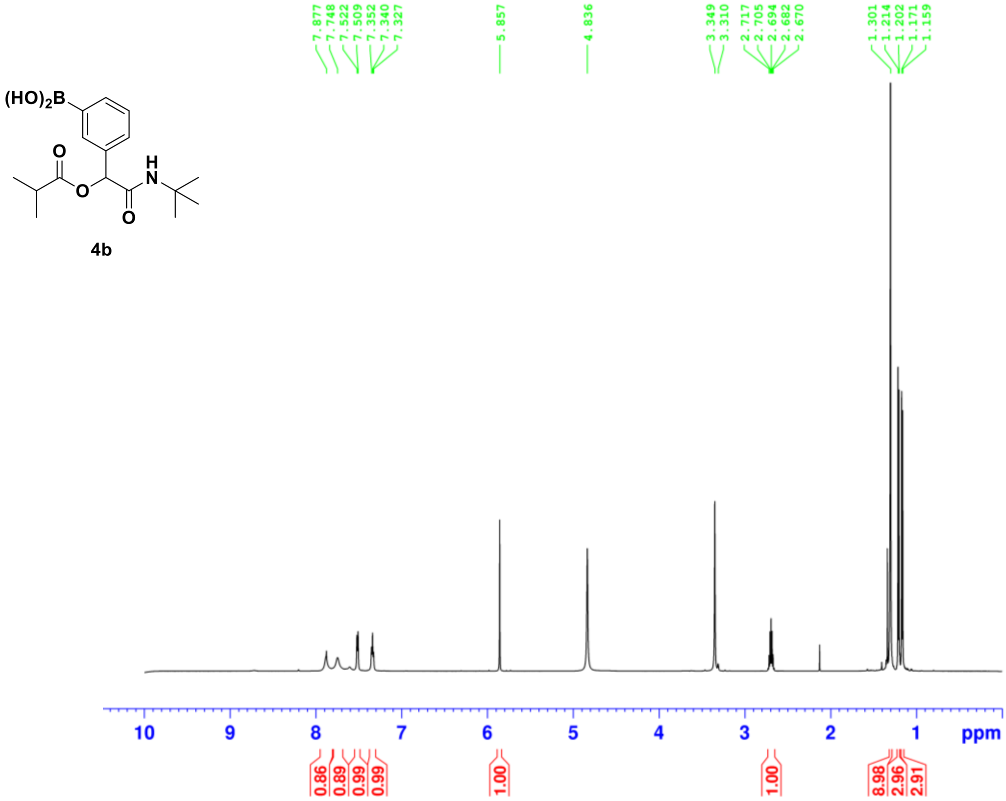

Figure S5. The ^{1}H NMR spectrum of **4b** in CD_3OD

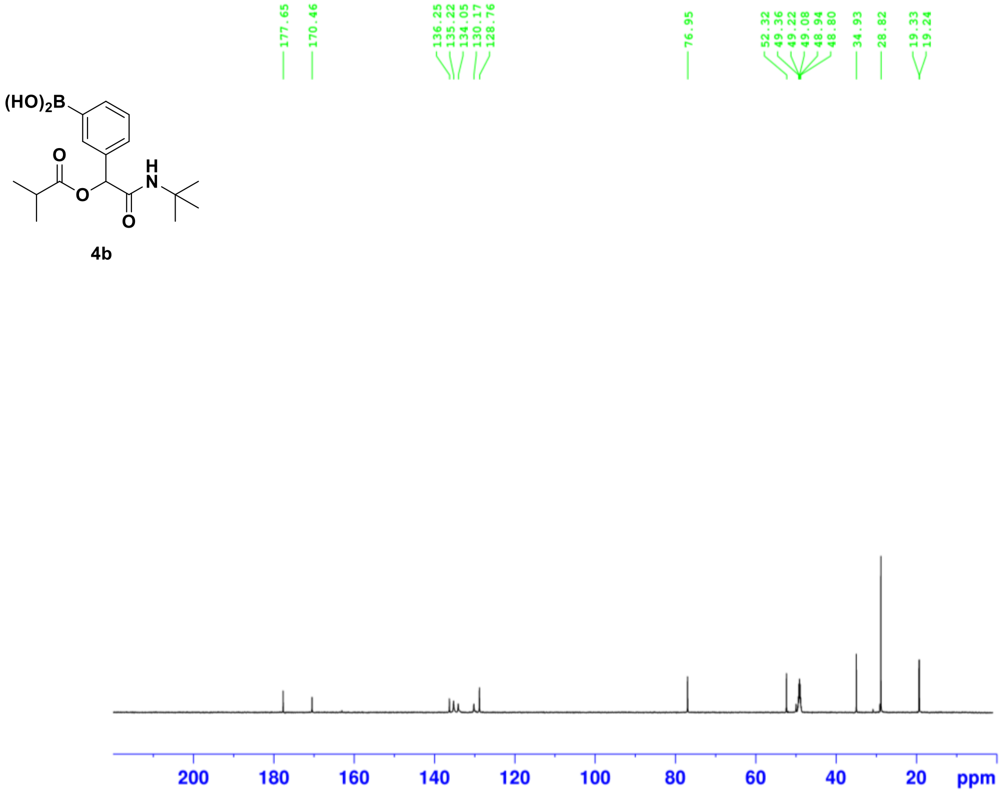

Figure S6. The ^{13}C NMR spectrum of **4b** in CD_3OD

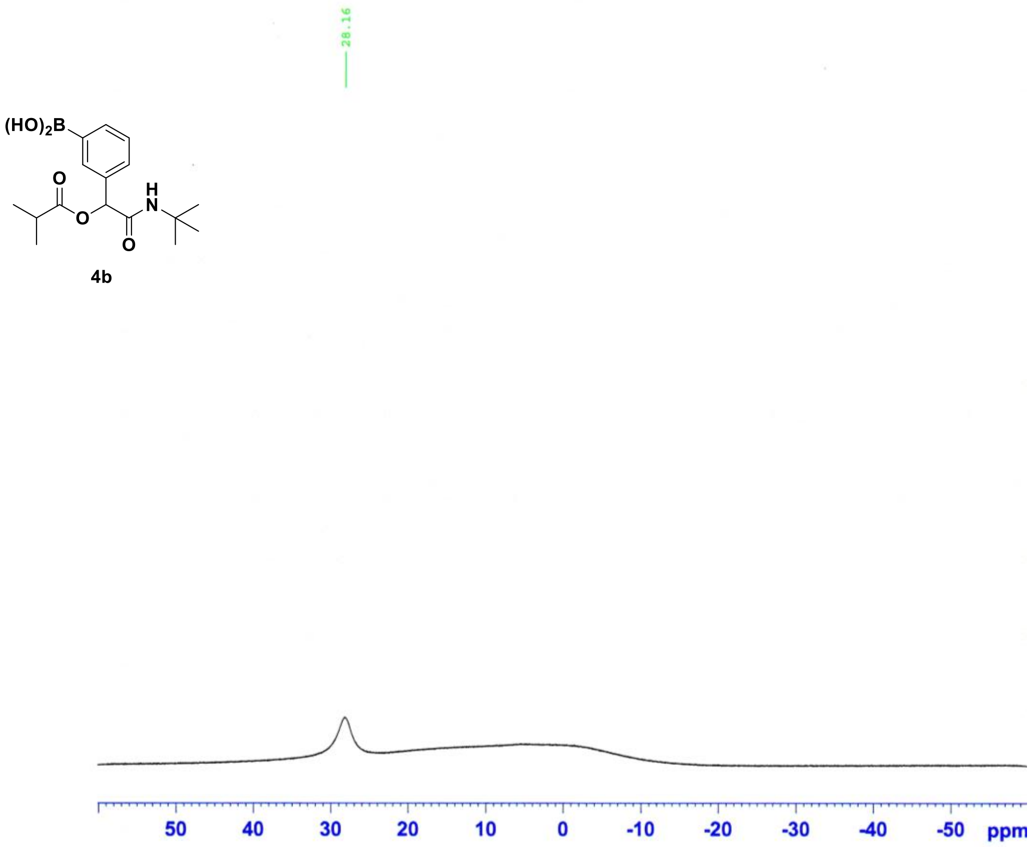

Figure S7. The ^{11}B NMR spectrum of **4b** in CD_3OD

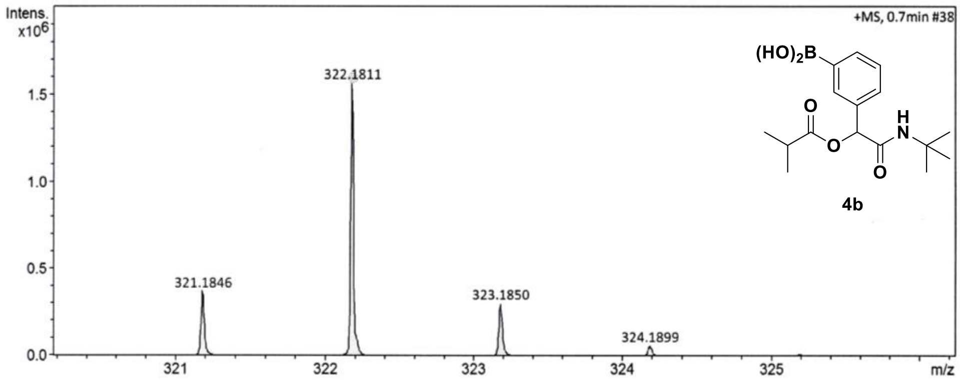

Figure S8. The HRMS (ESI+) of spectrum of **4b**

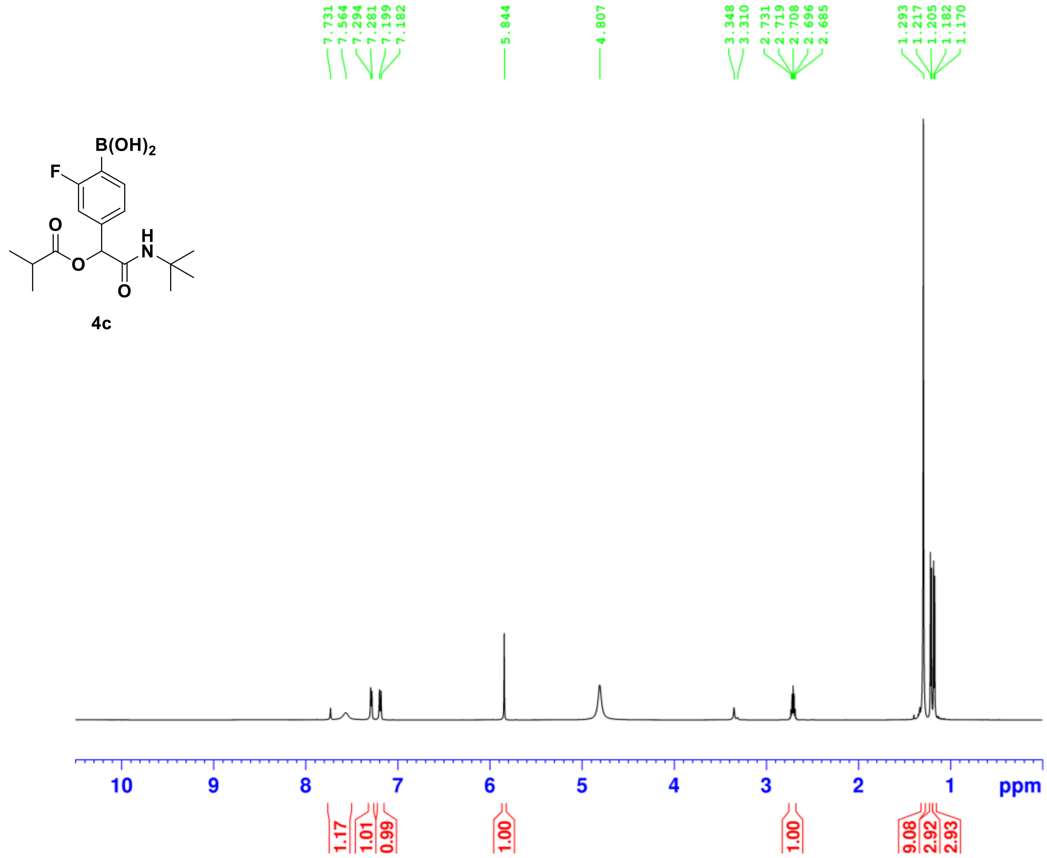

Figure S9. The ¹H NMR spectrum of **4c** in CD3OD

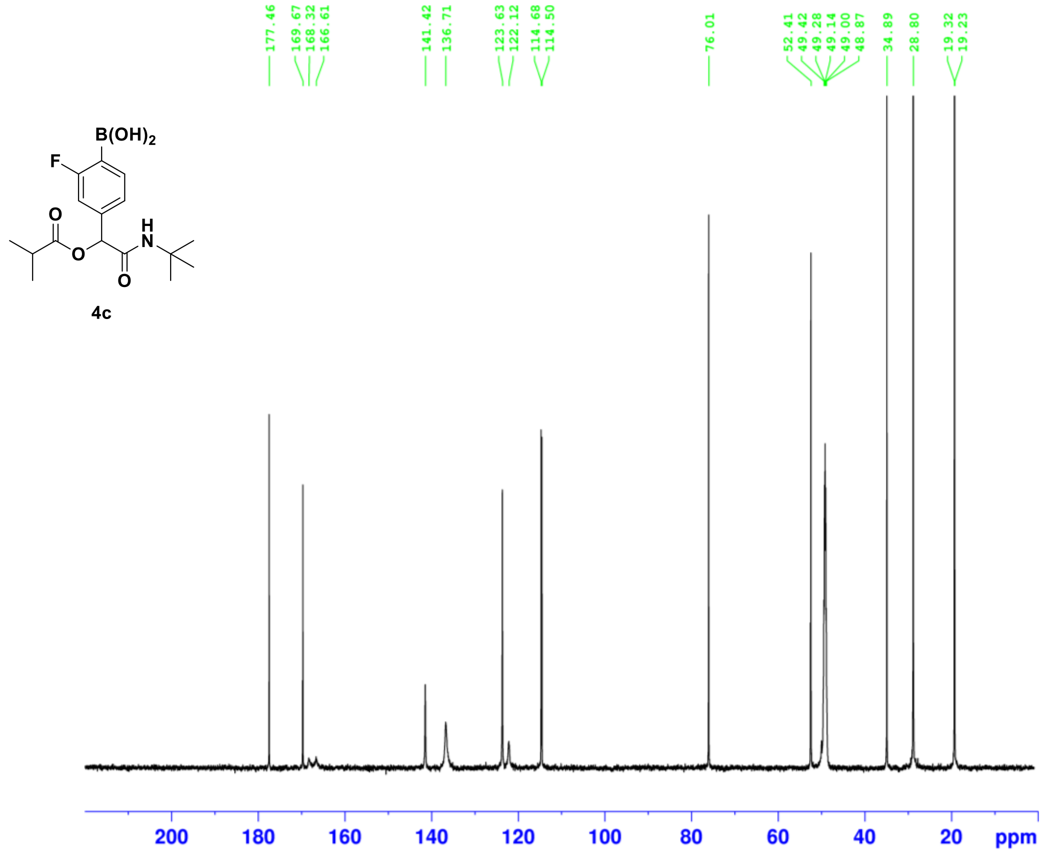

Figure S10. The ¹³C NMR pectrum of **4c** in CD3OD

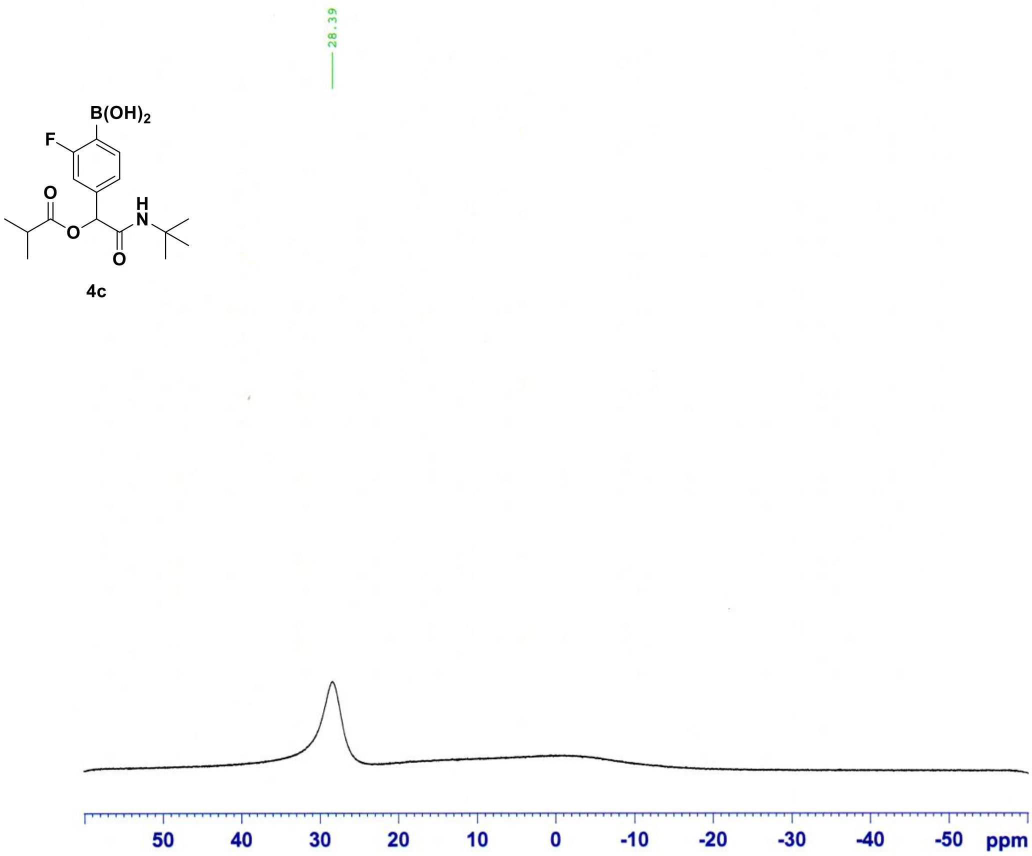

Figure S11. The ^{11}B NMR spectrum of **4c** in CD_3OD

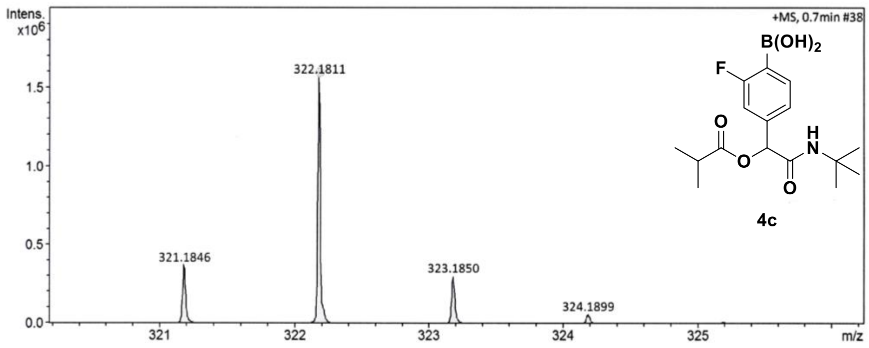

Figure S12. The HRMS (ESI+) of spectrum of **4c**

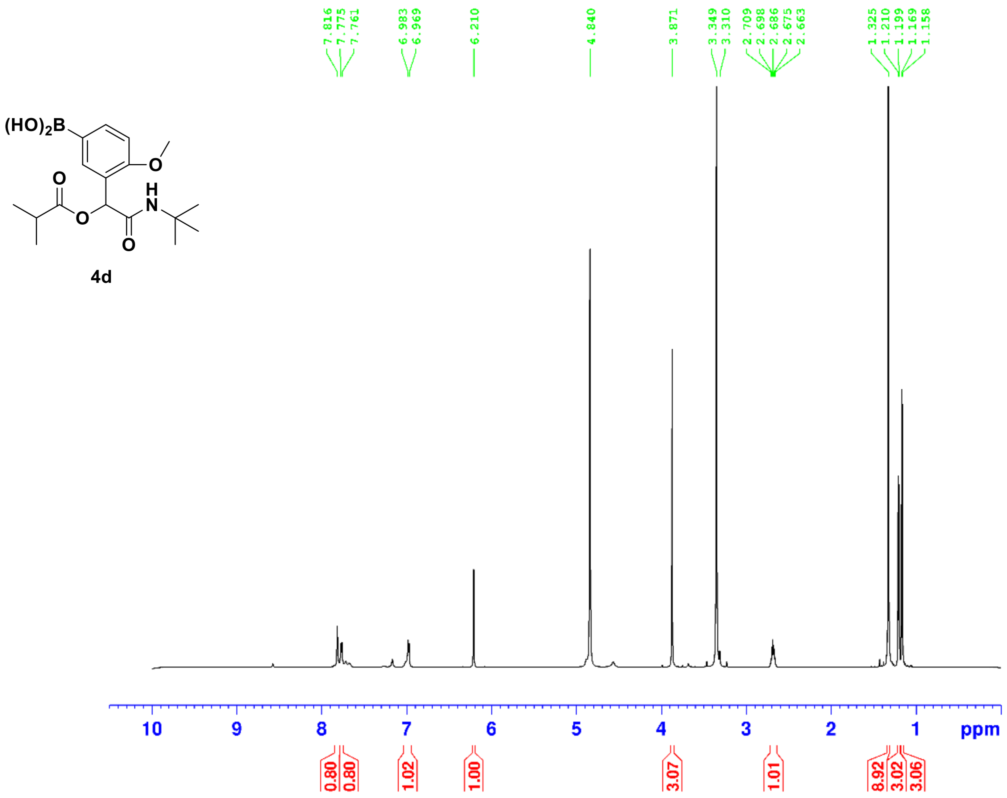

Figure S13. The ^{1}H NMR spectrum of **4d** in CD_3OD

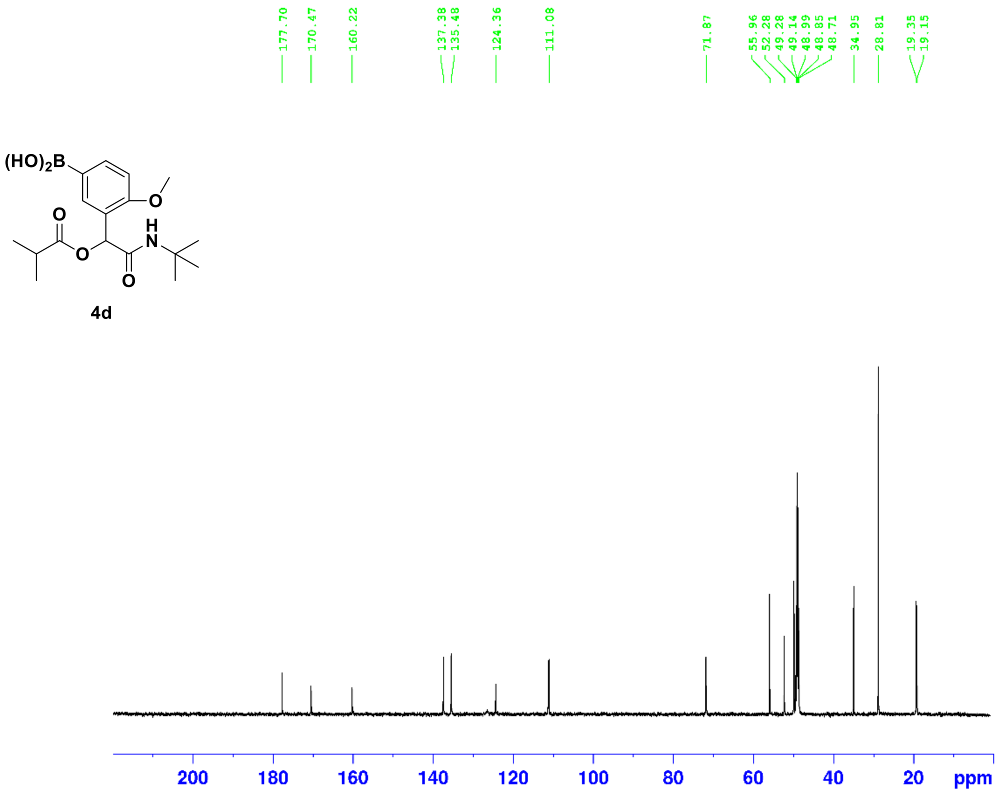

Figure S14. The ^{13}C NMR spectrum of **4d** in CD_3OD

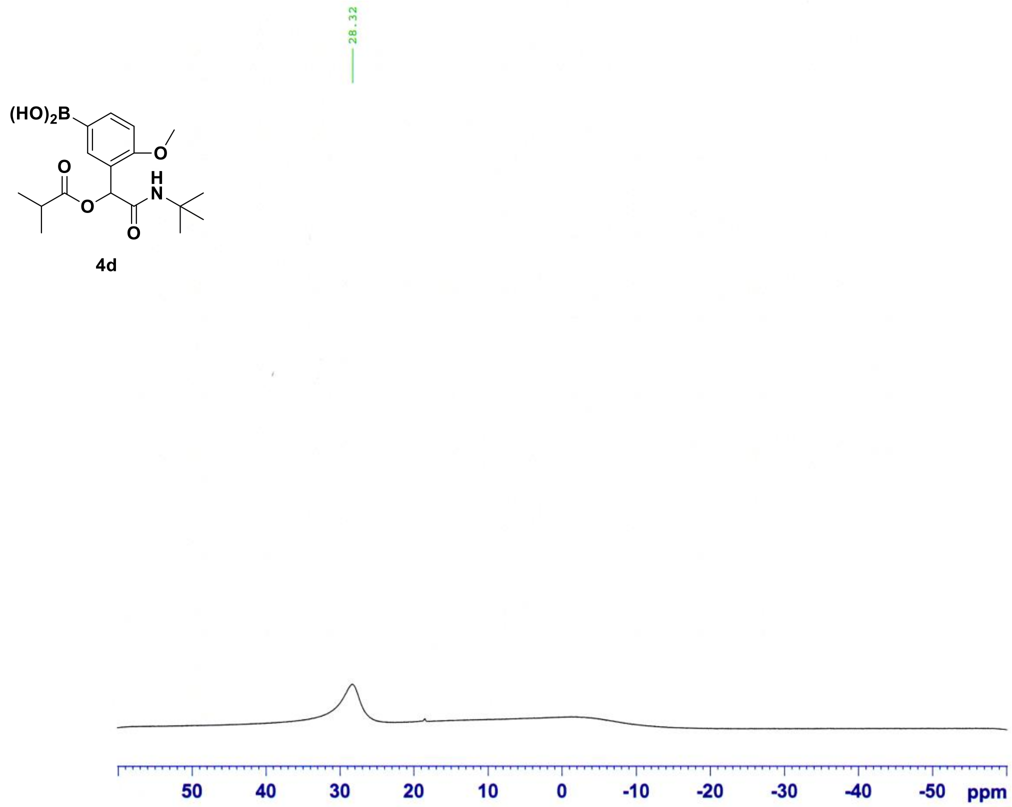

Figure S15. The ^{11}B NMR spectrum of **4d** in CD_3OD

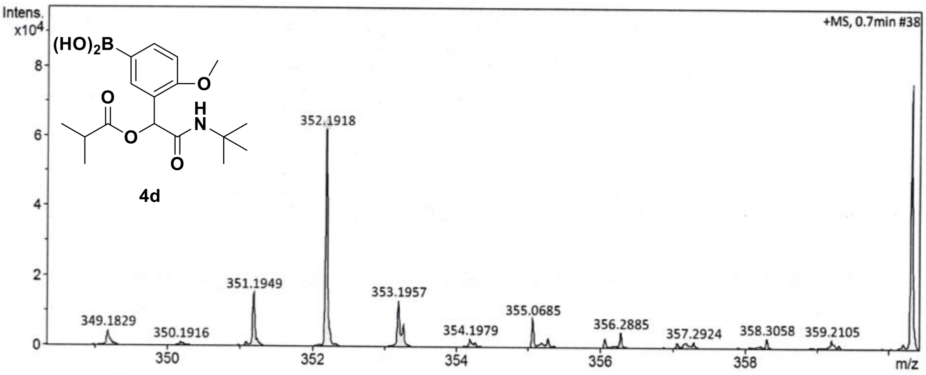

Figure S16. The HRMS (ESI^+) of spectrum of **4d**

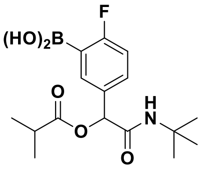

4e

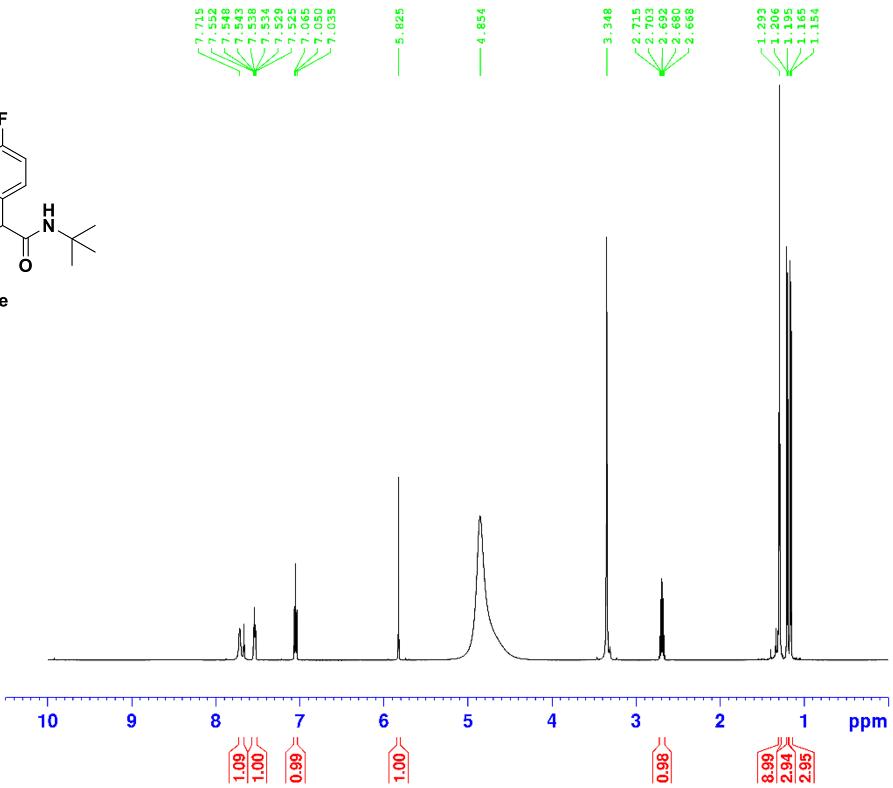

Figure S17. The ^{1}H NMR spectrum of **4e** in CD_3OD

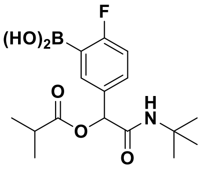

4e

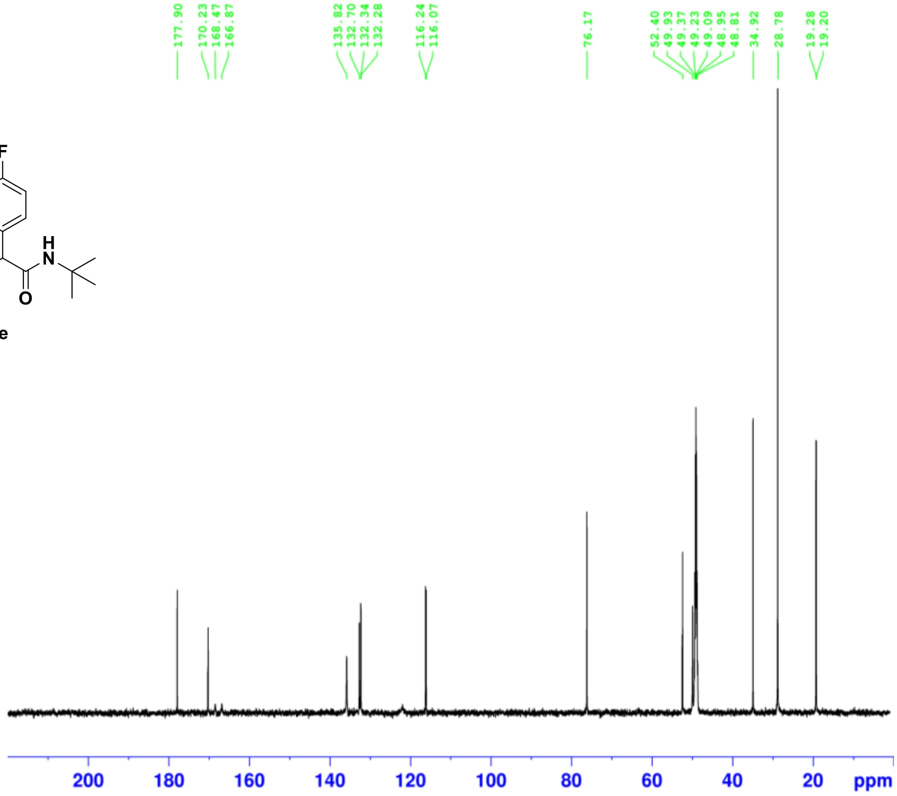

Figure S18. The ^{13}C NMR spectrum of **4e** in CD_3OD

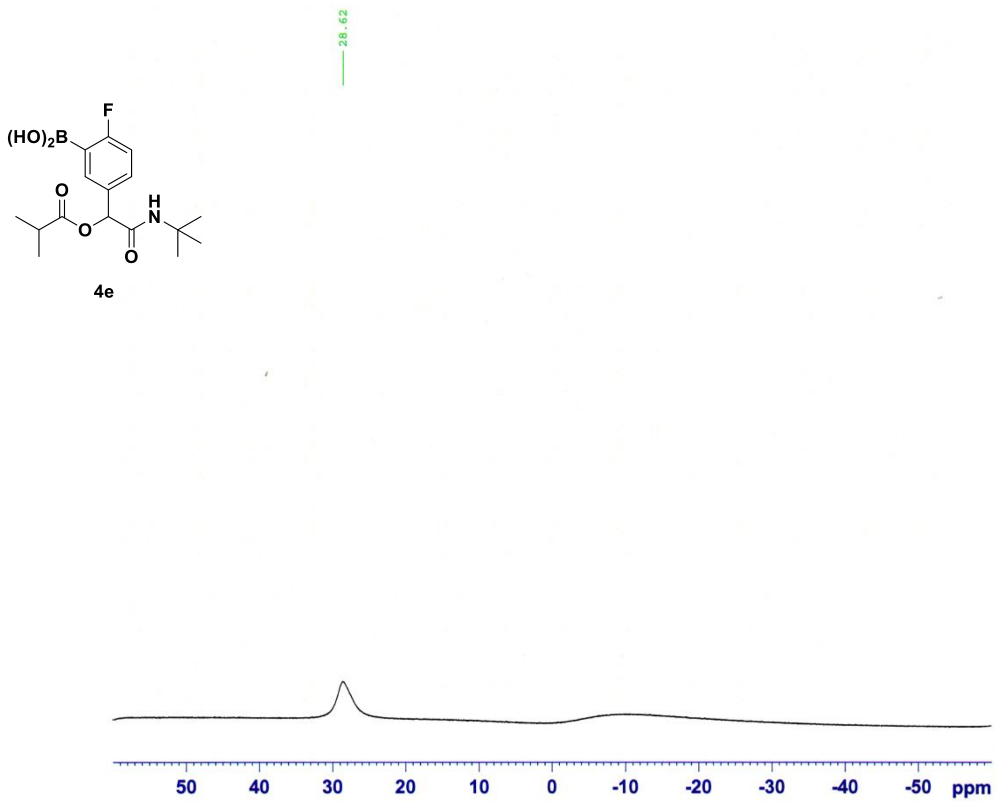

Figure S19. The ^{11}B NMR spectrum of **4e** in CD_3OD

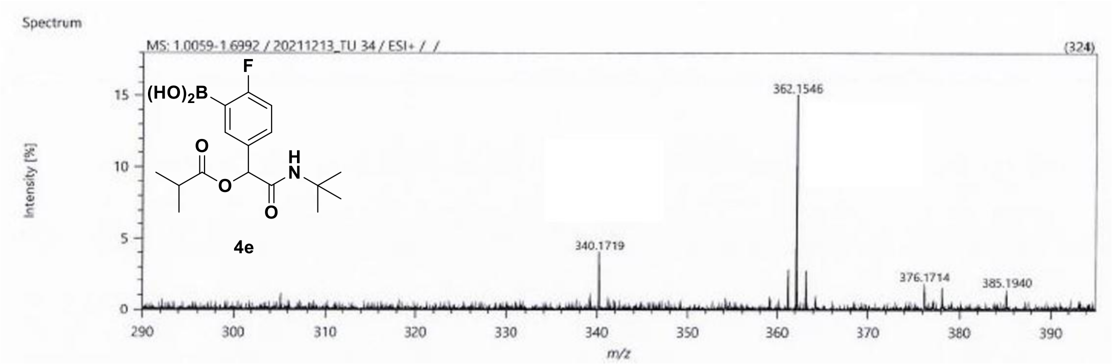

Figure S20. The HRMS (ESI+) of spectrum of **4e**

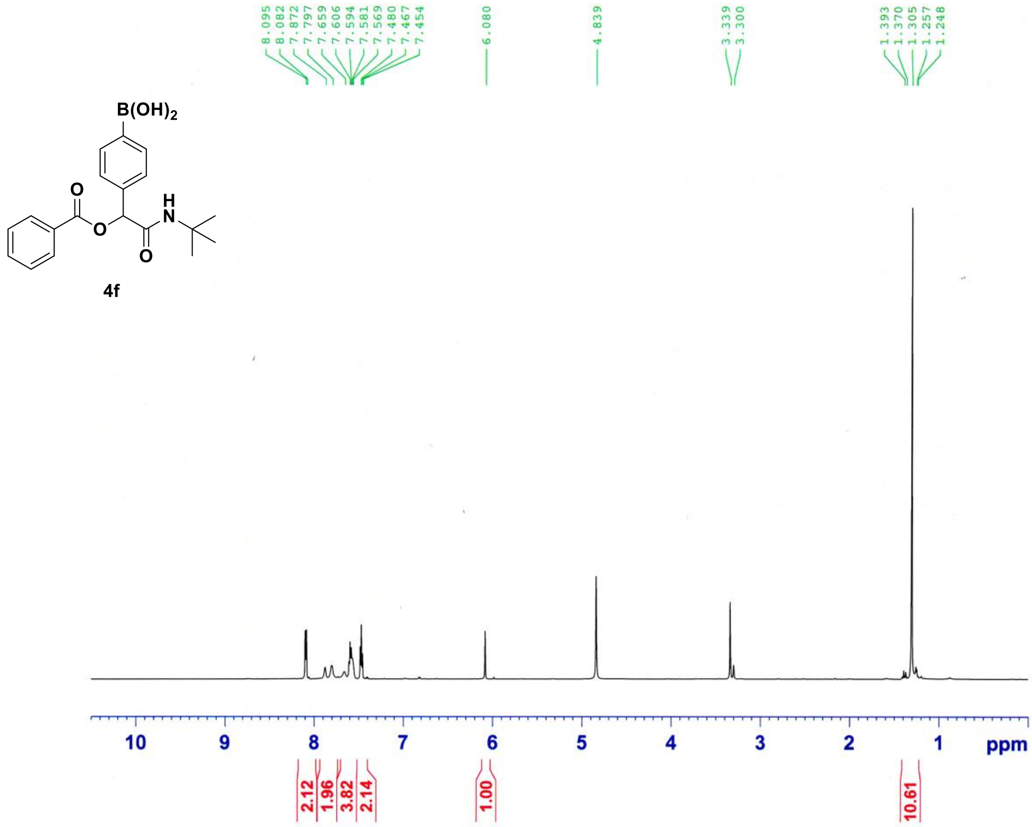

Figure S21. The ^{1}H NMR spectrum of **4f** in CD_3OD

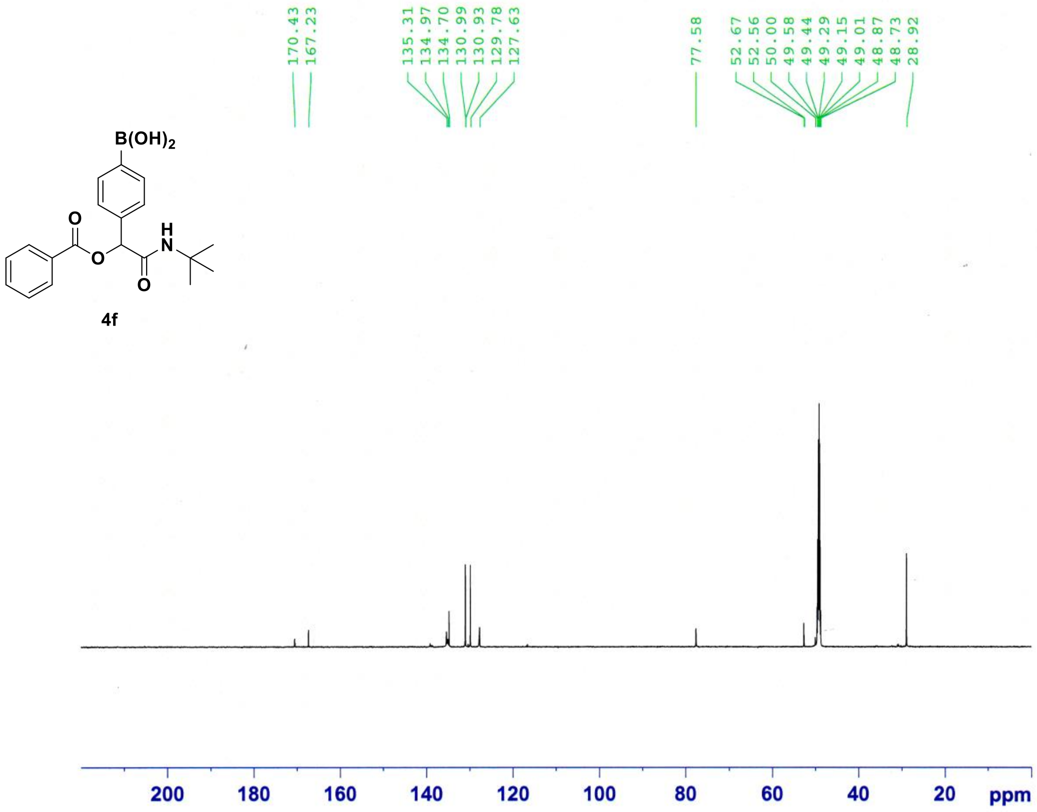

Figure S22. The ^{13}C NMR spectrum of **4f** in CD_3OD

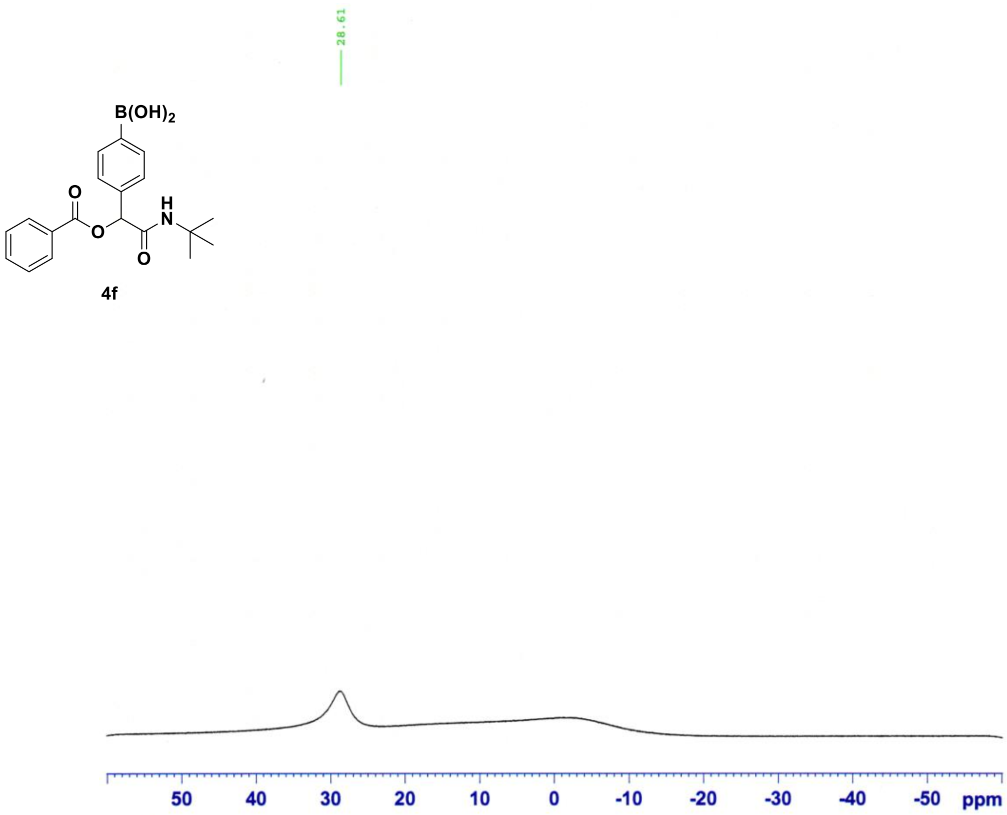

Figure S23. The ^{11}B NMR spectrum of **4f** in CD_3OD

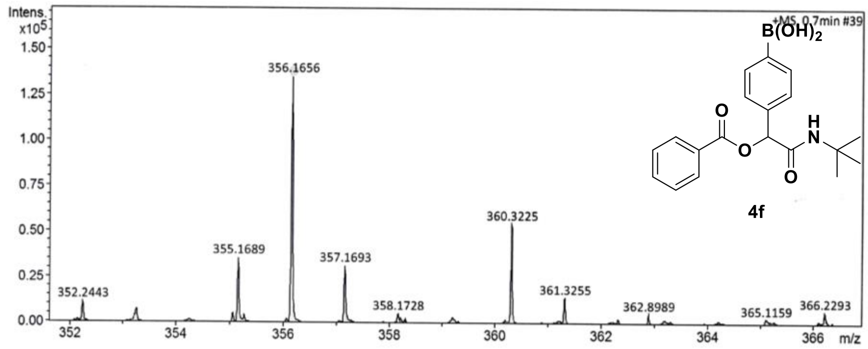

Figure S24. The HRMS (ESI+) of spectrum of **4f**

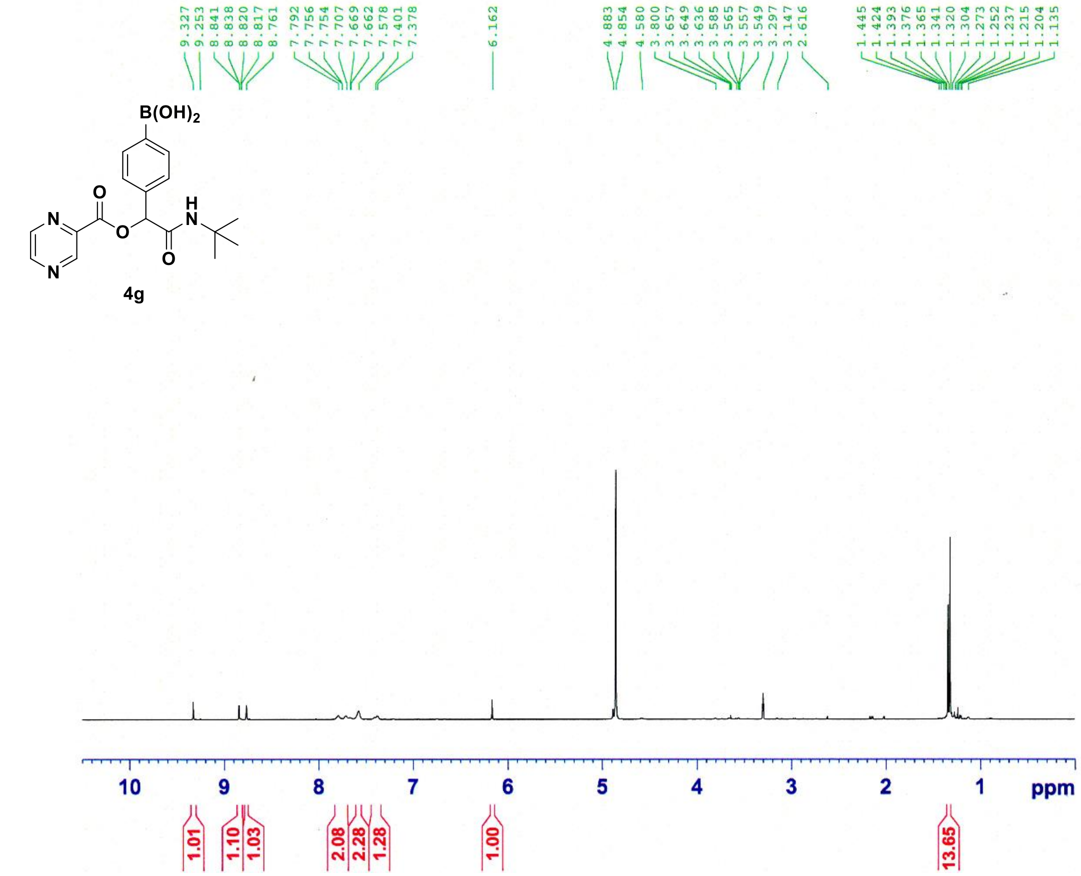

Figure S25. The ^{1}H NMR spectrum of **4g** in CD_3OD

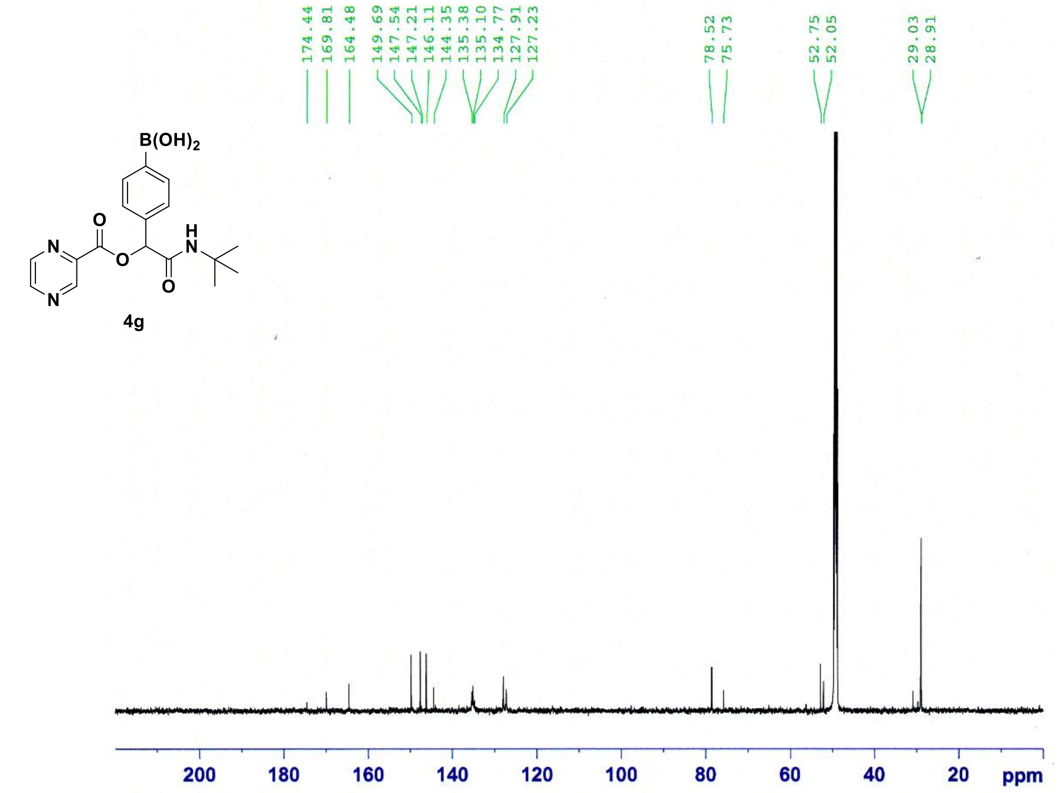

Figure S26. The ^{13}C NMR spectrum of **4g** in CD_3OD

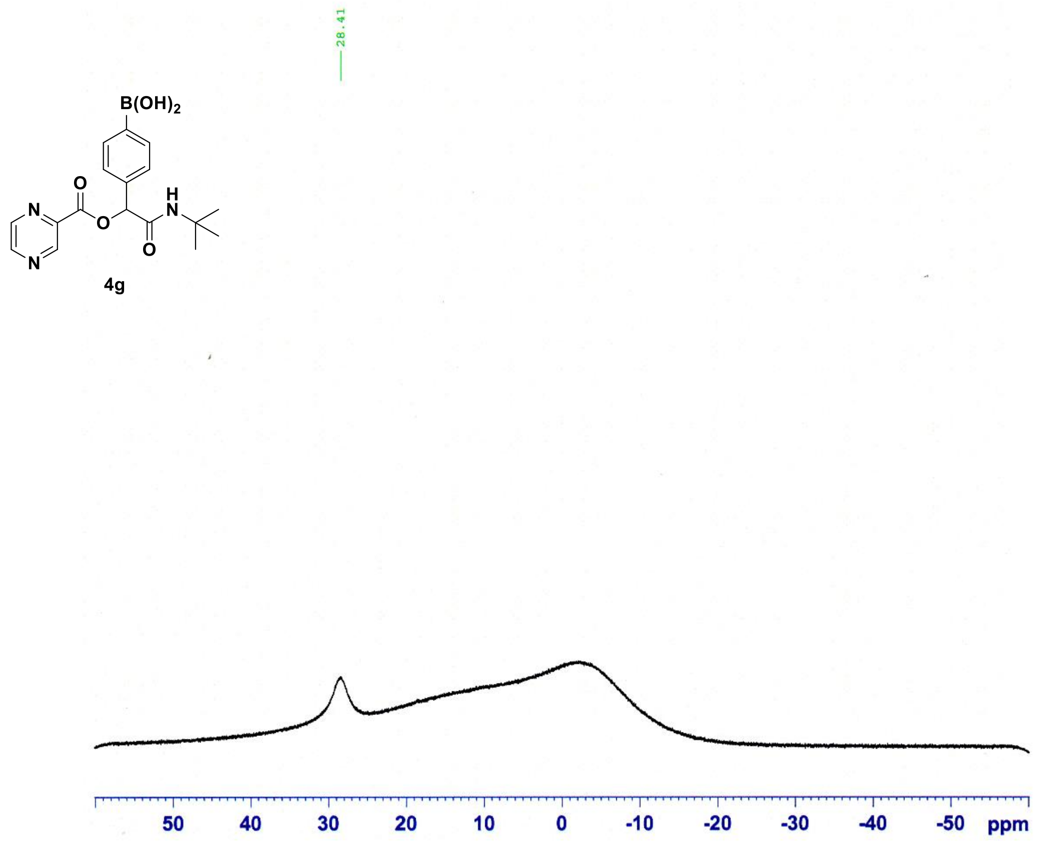

Figure S27. The ^{11}B NMR spectrum of **4g** in CD_3OD

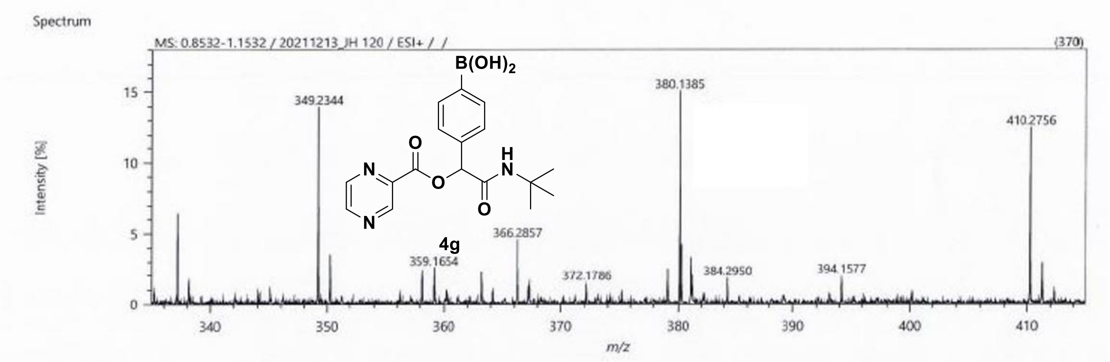

Figure S28. The HRMS (ESI$^+$) of spectrum of **4g**

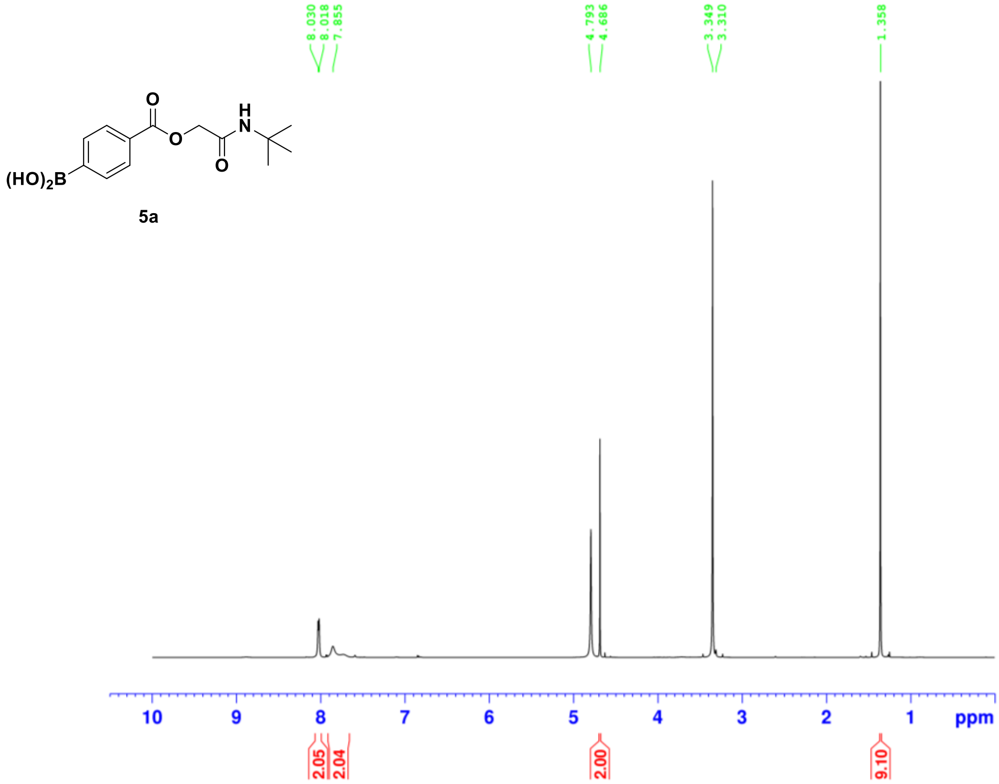

Figure S29. The ^{1}H NMR spectrum of **5a** in CD3OD

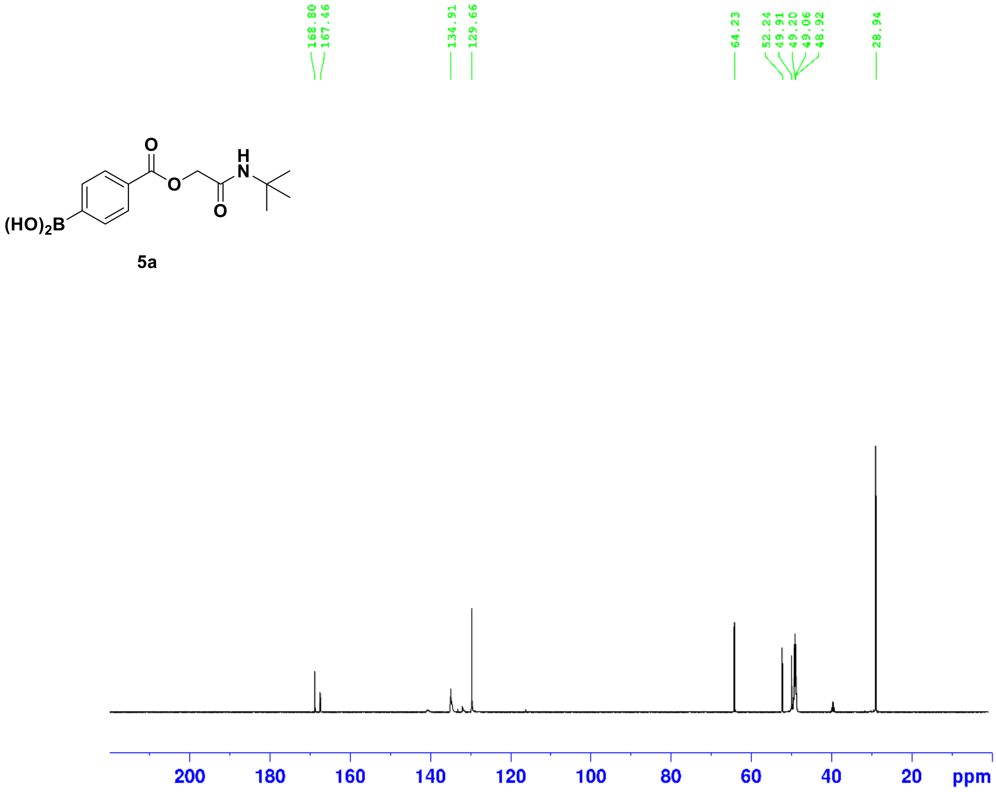

Figure S30. The ^{13}C NMR spectrum of **5a** in CD3OD

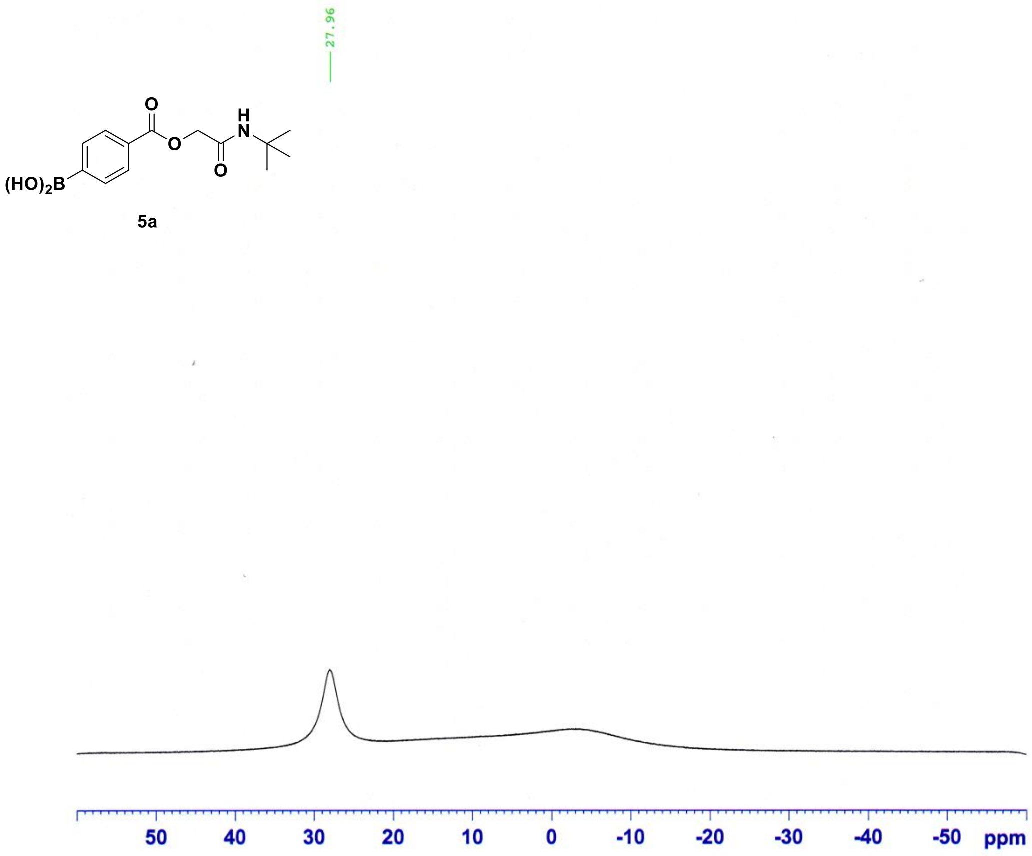

Figure S31. The ^{11}B NMR spectrum of **5a** in CD_3OD

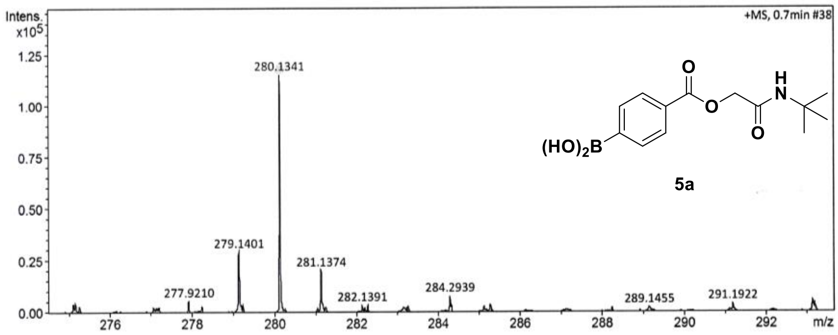

Figure S32. The HRMS (ESI+) of spectrum of **5a**

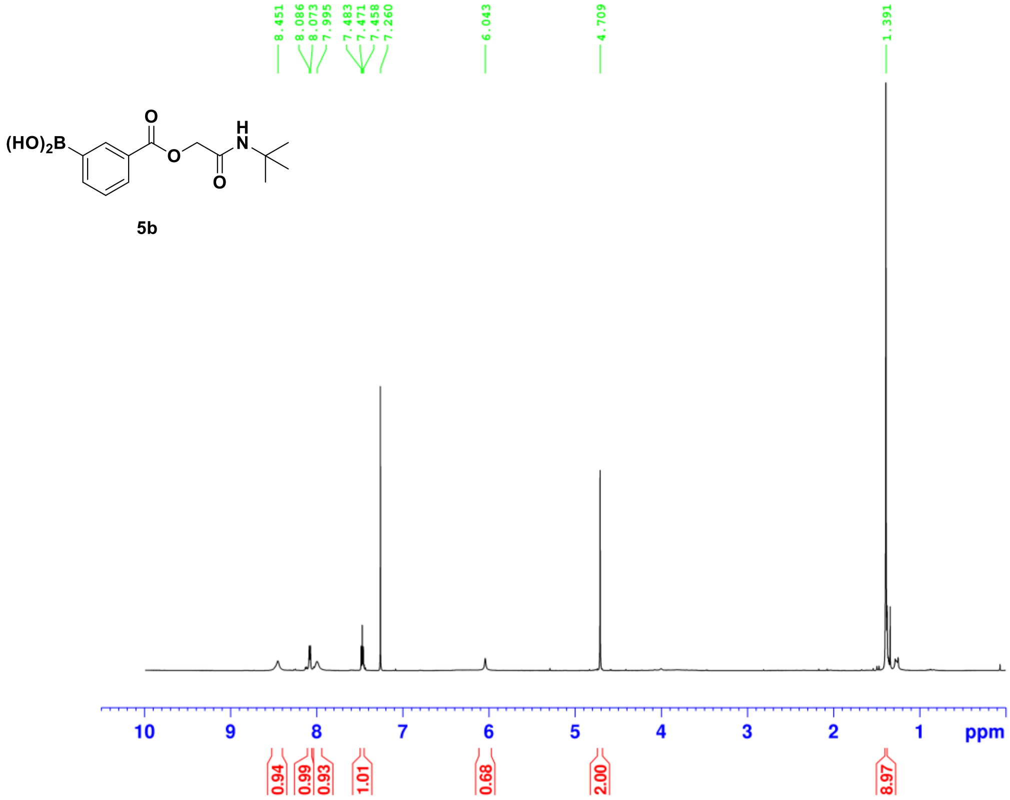

Figure S33. The ^{1}H NMR spectrum of **5b** in CD_3OD

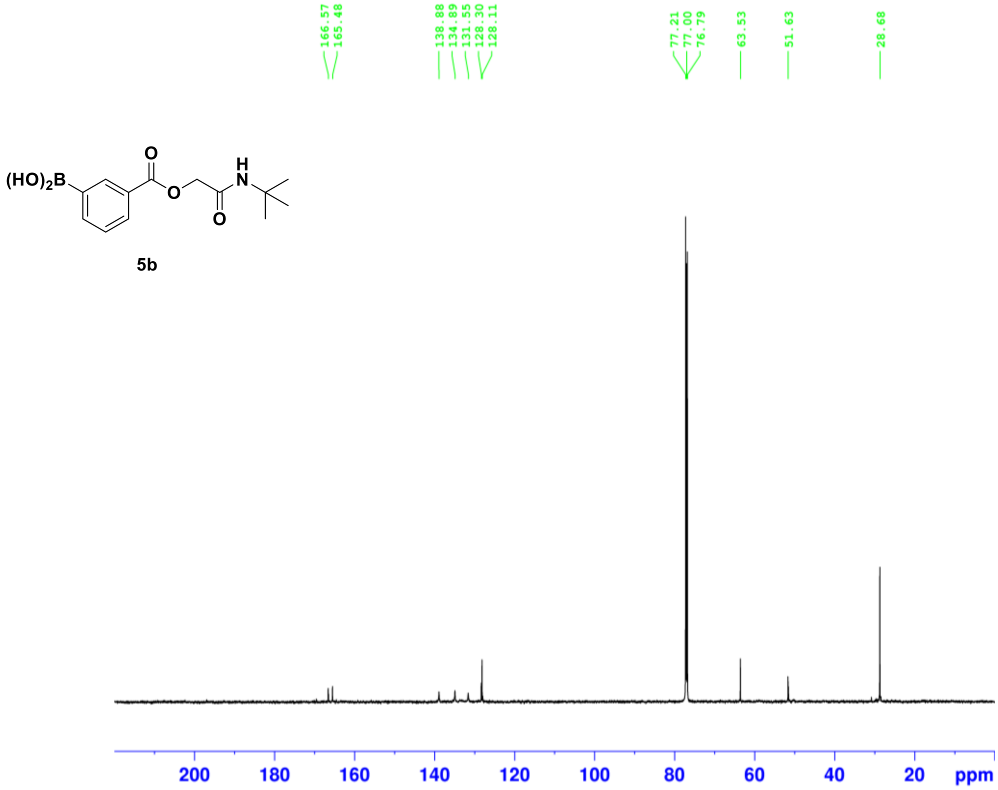

Figure S34. The ^{13}C NMR spectrum of **5b** in CD_3OD

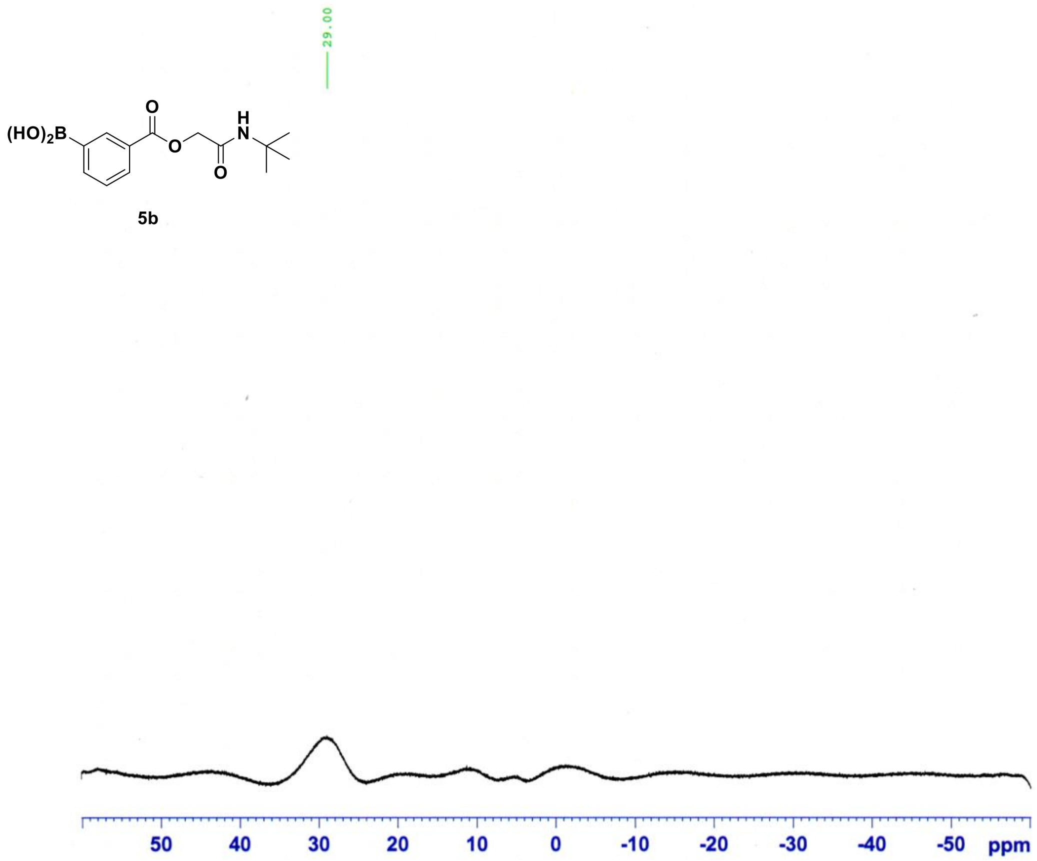

Figure S35. The ^{11}B NMR spectrum of **5b** in CD_3OD

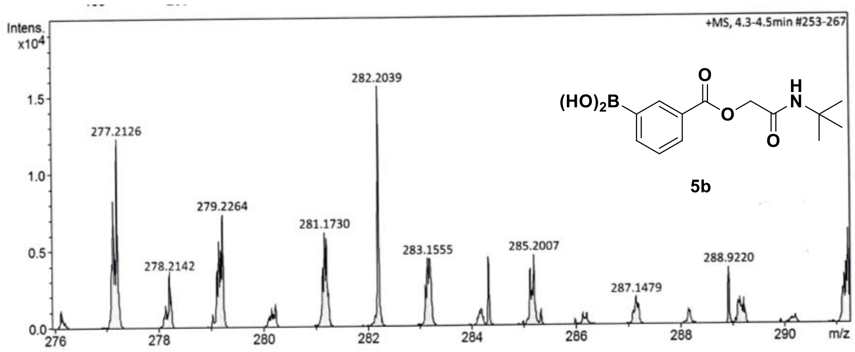

Figure S36. The HRMS (ESI^+) of spectrum of **5b**

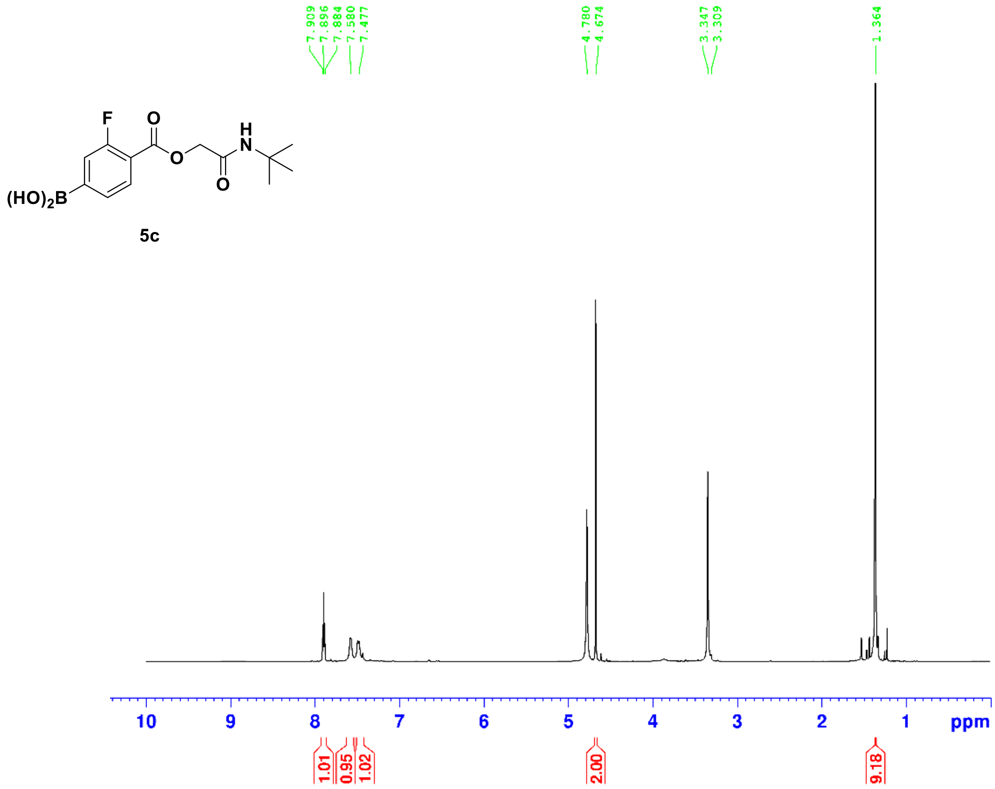

Figure S37. The ^{1}H NMR spectrum of **5c** in CD_3OD

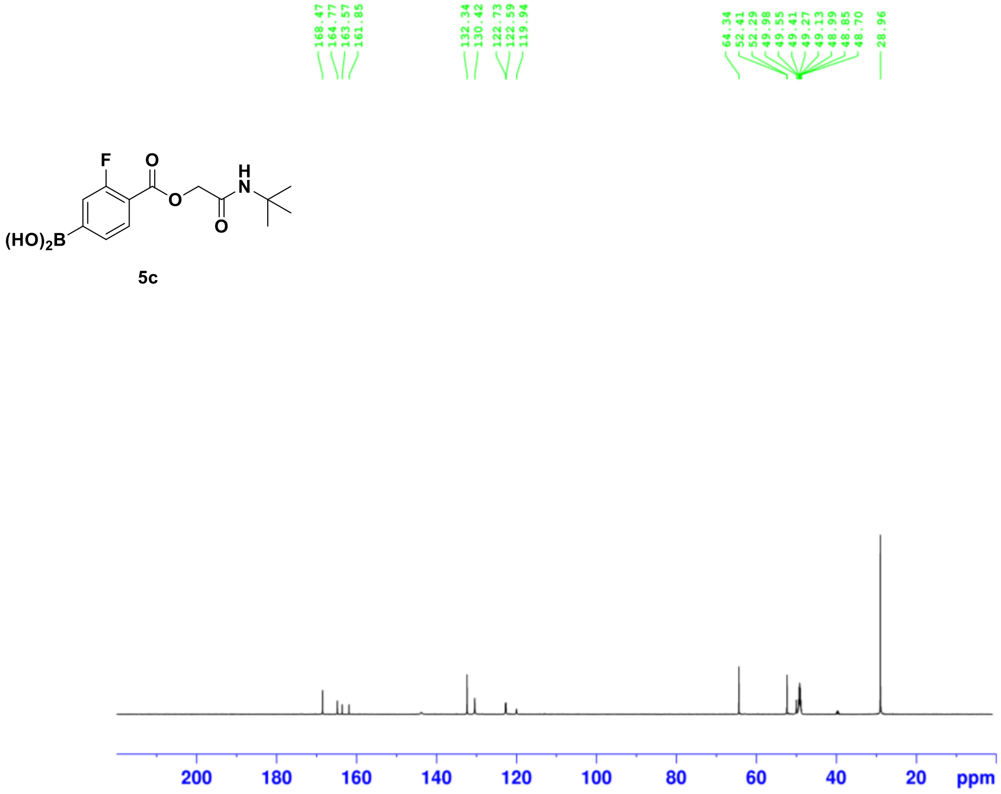

Figure S38. The ^{13}C NMR spectrum of **5c** in CD_3OD

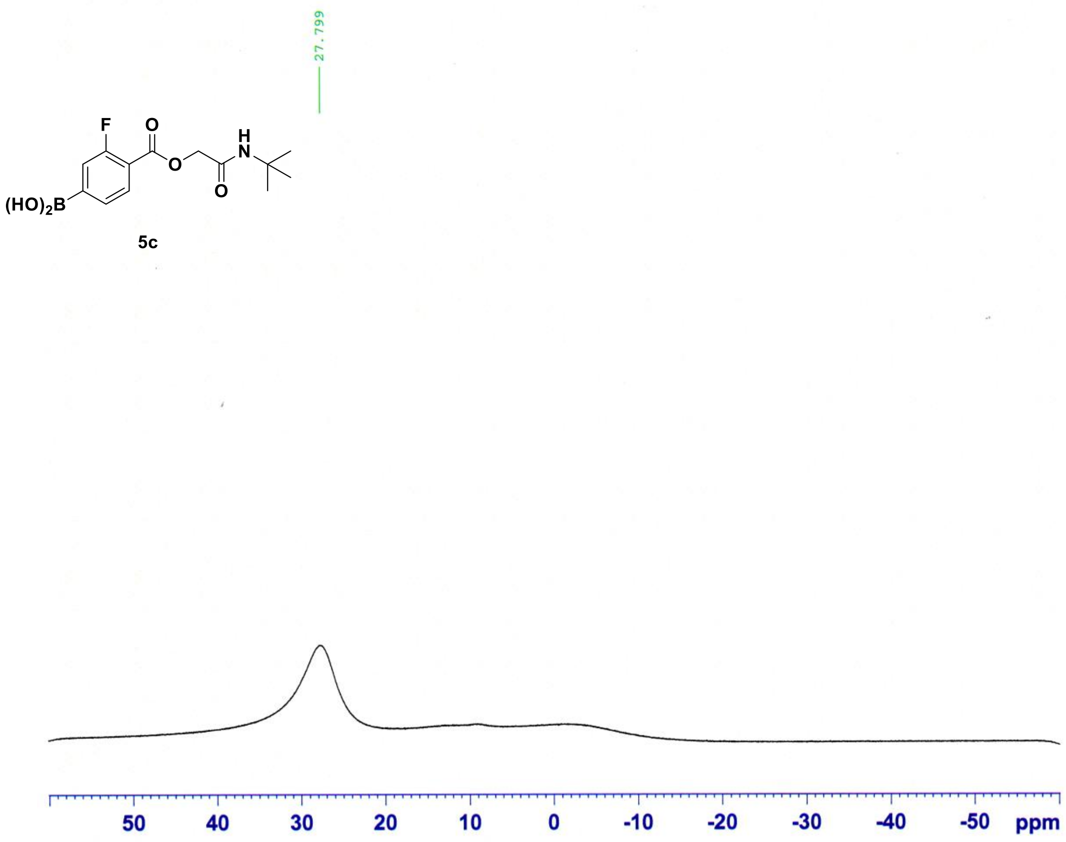

Figure S39. The ^{11}B NMR spectrum of **5c** in CD_3OD

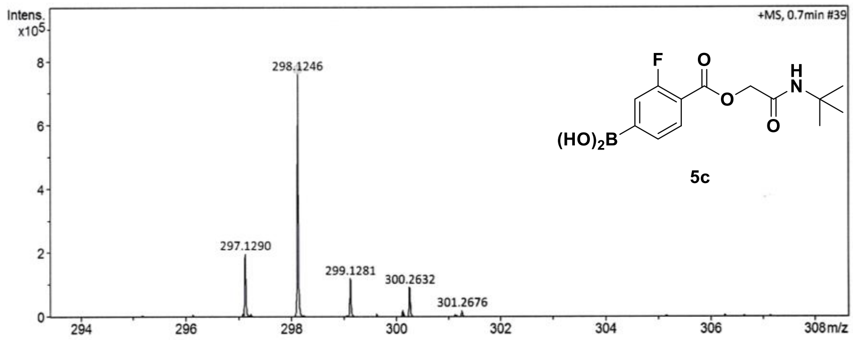

Figure S40. The HRMS (ESI+) of spectrum of **5c**

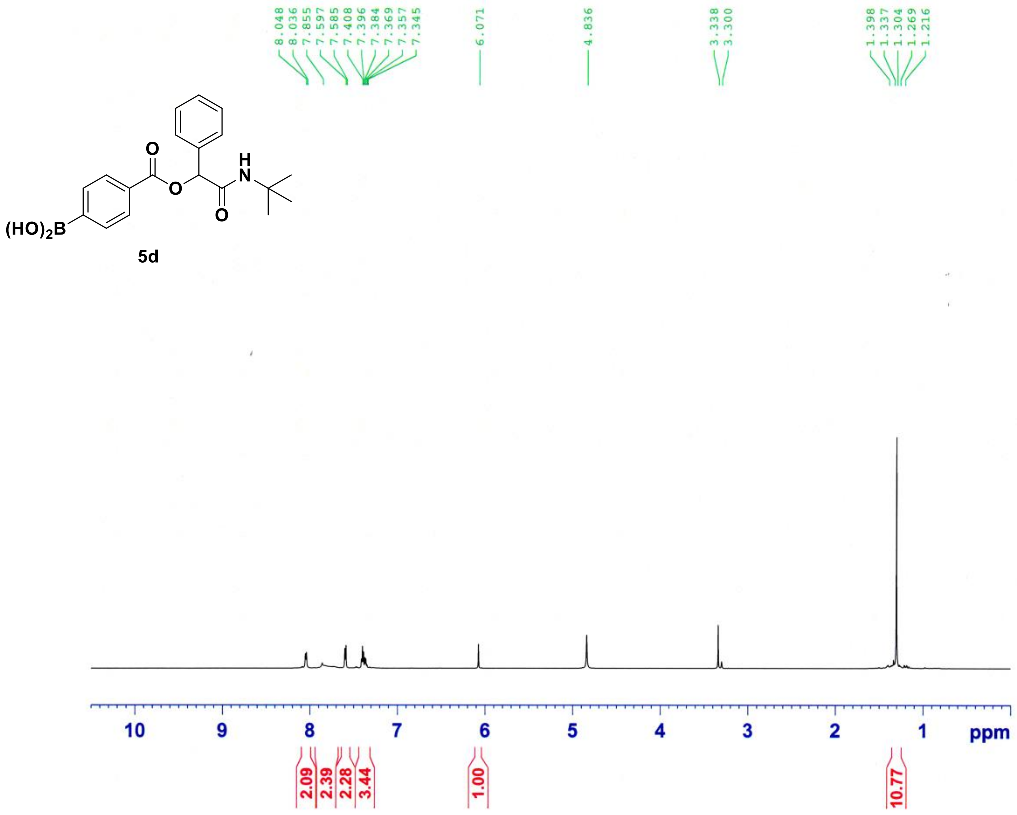

Figure S41. The ^{1}H NMR spectrum of **5d** in CD_3OD

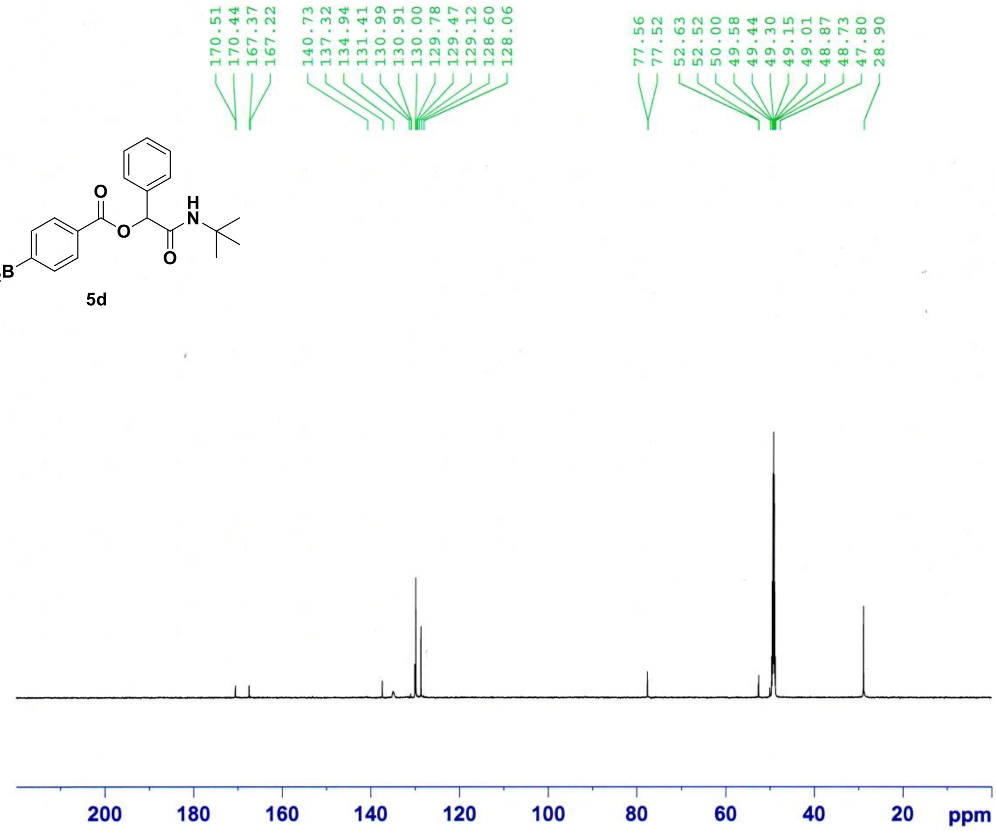

Figure S42. The ^{13}C NMR spectrum of **5d** in CD_3OD

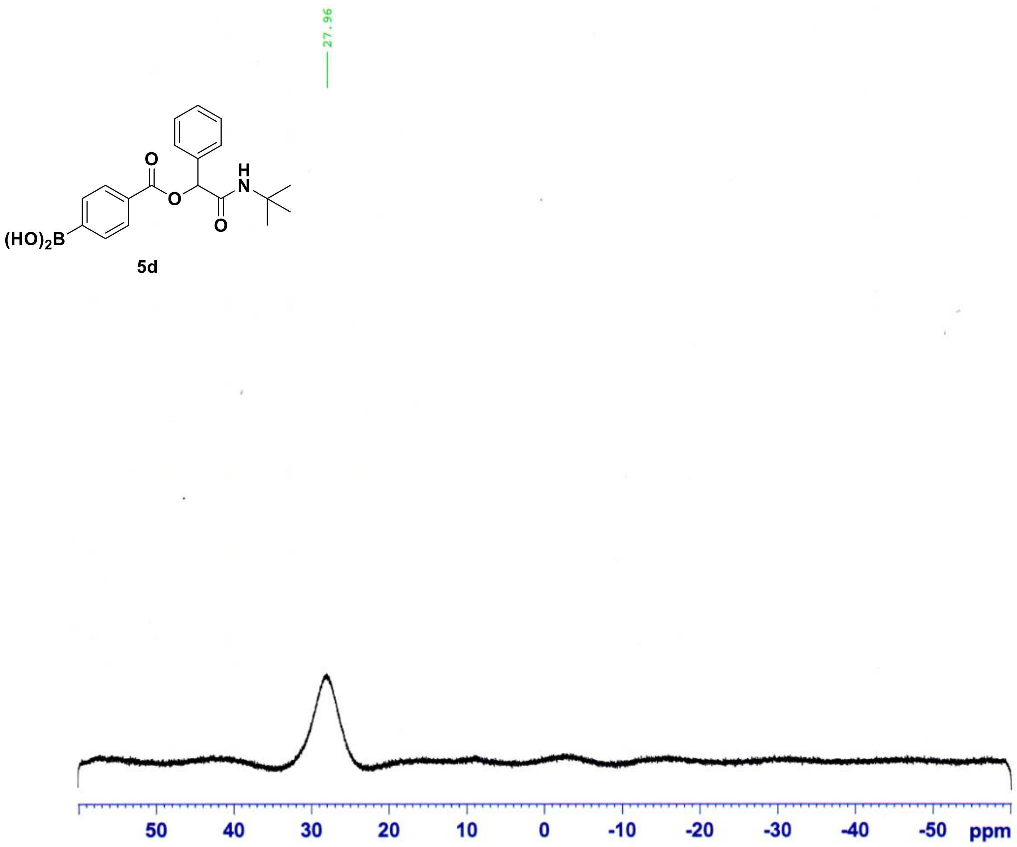

Figure S43. The ^{11}B NMR spectrum of **5d** in CD_3OD

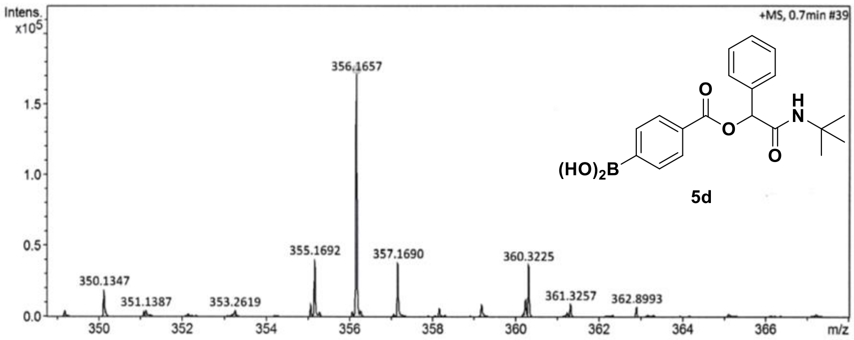

Figure S44. The HRMS (ESI$^+$) of spectrum of **5d**

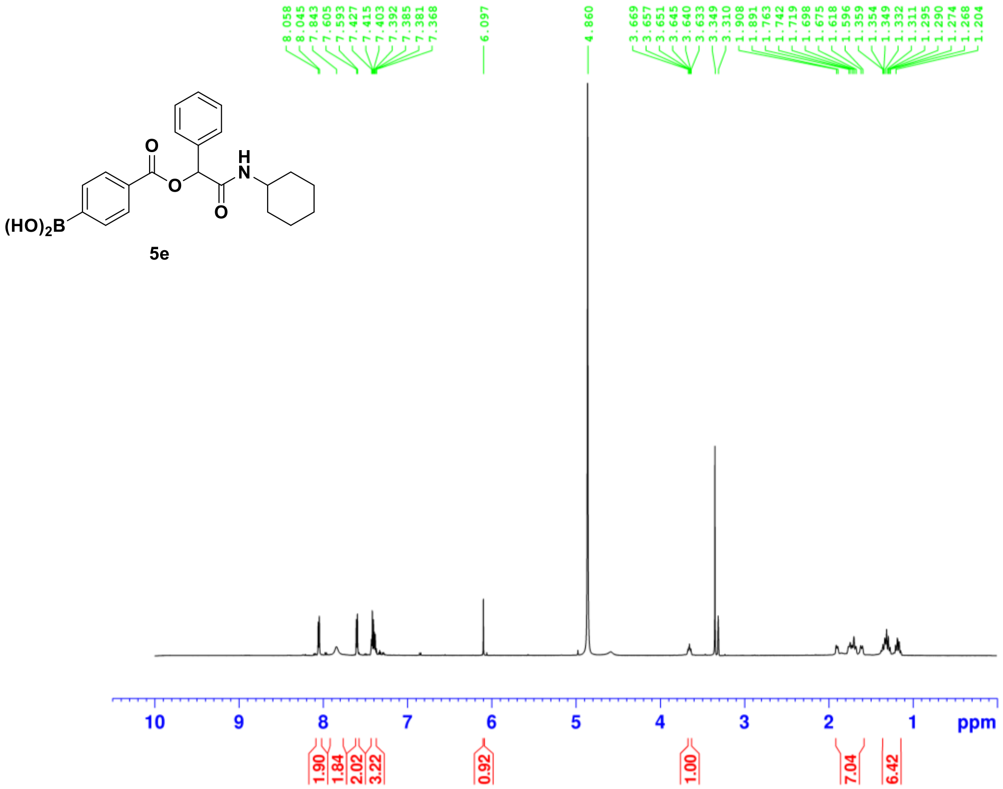

Figure S45. The ^{1}H NMR spectrum of **5e** in CD_3OD

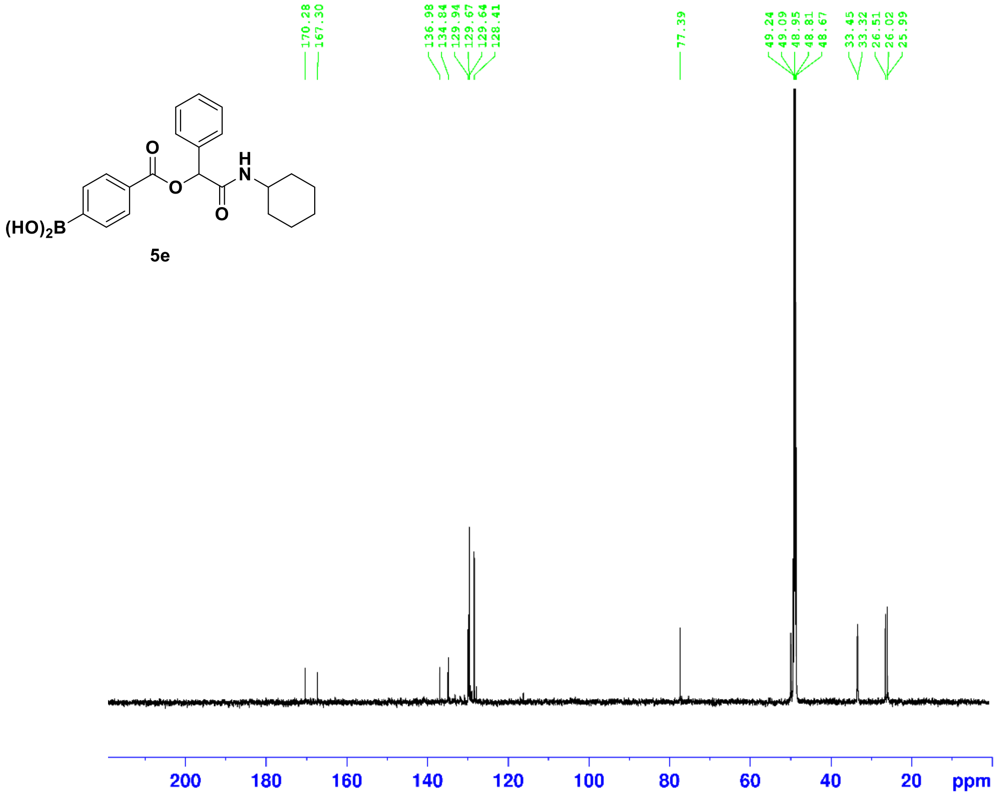

Figure S46. The ^{13}C NMR spectrum of **5e** in CD_3OD

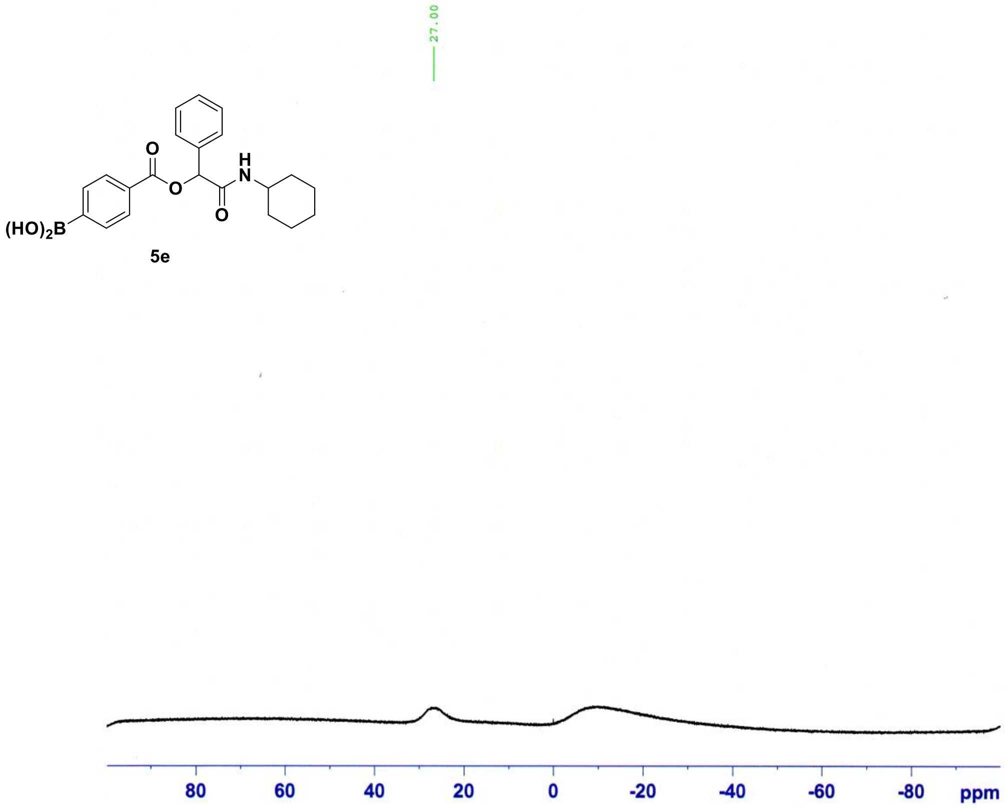

Figure S47. The ¹¹B NMR spectrum of **5e** in CD_3OD

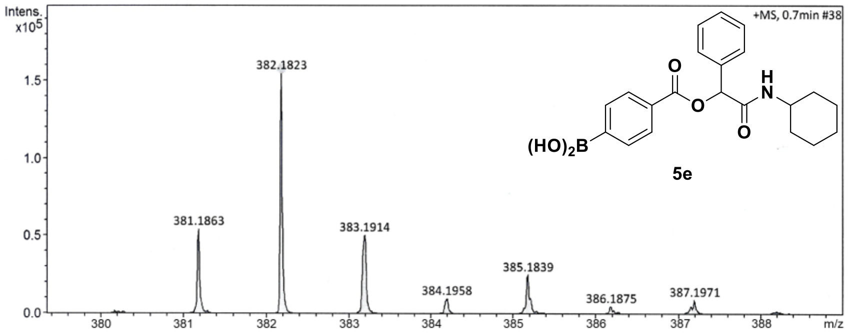

Figure S48. The HRMS (ESI+) of spectrum of **5e**

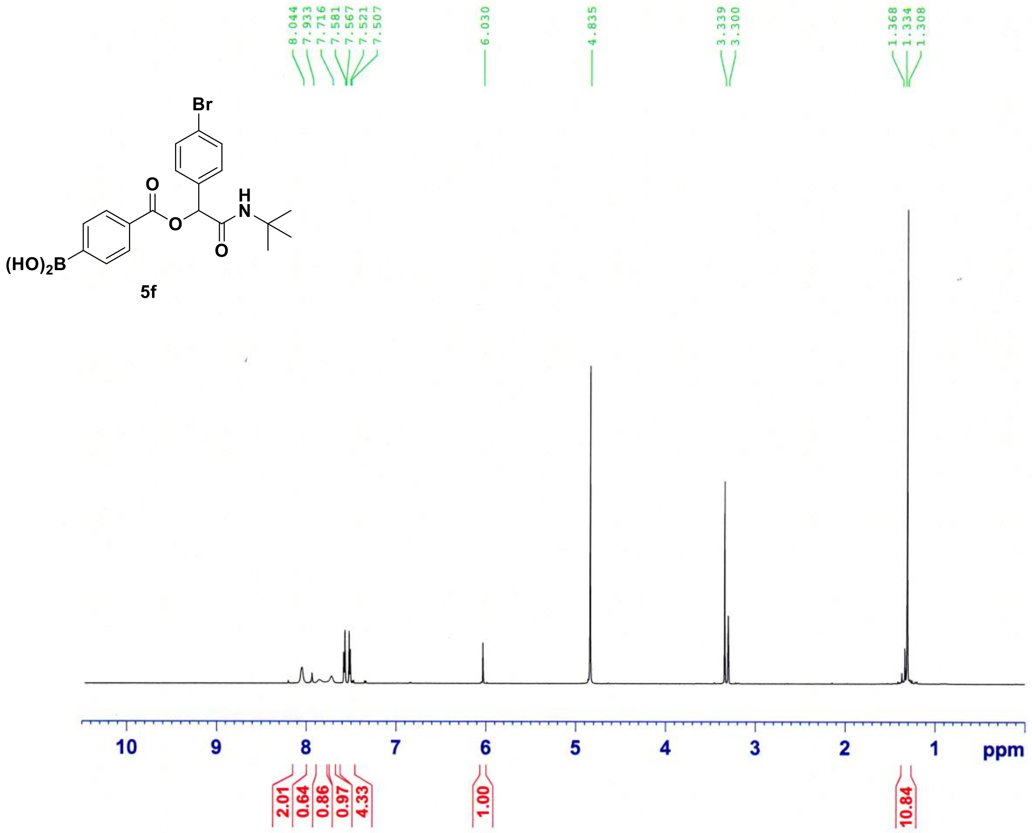

Figure S49. The ^{1}H NMR spectrum of **5f** in CD_3OD

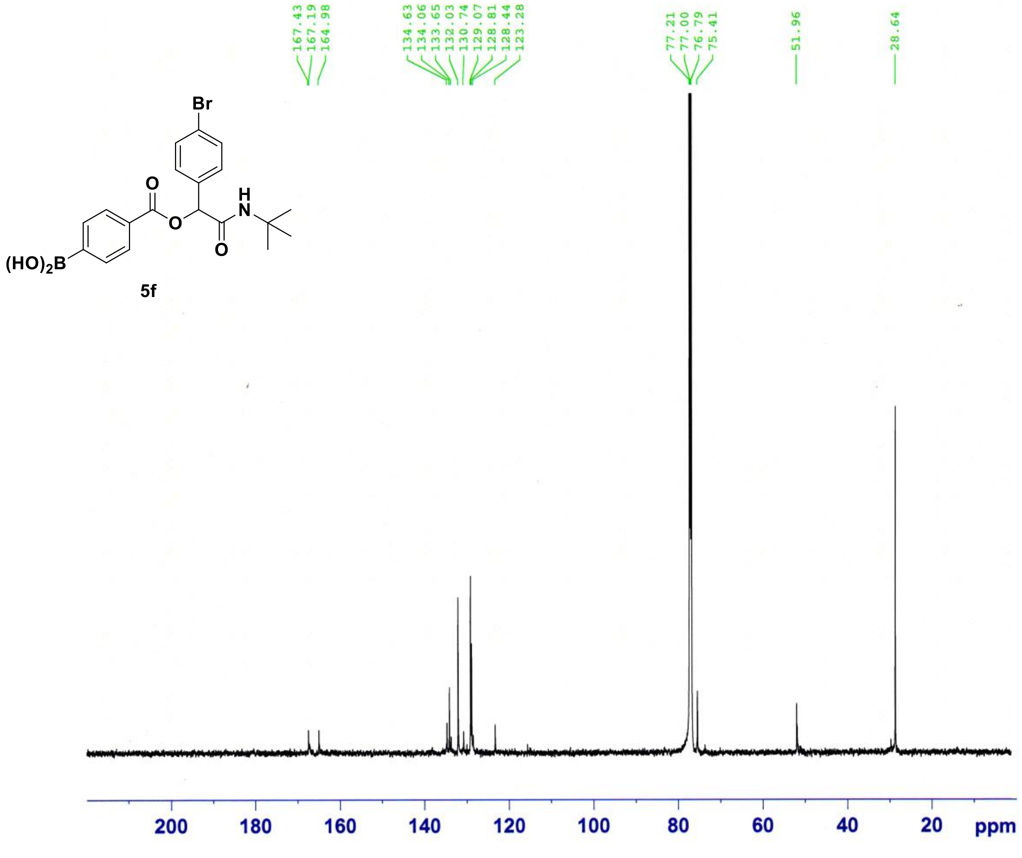

Figure S50. The ^{13}C NMR spectrum of **5f** in $CDCl_3$

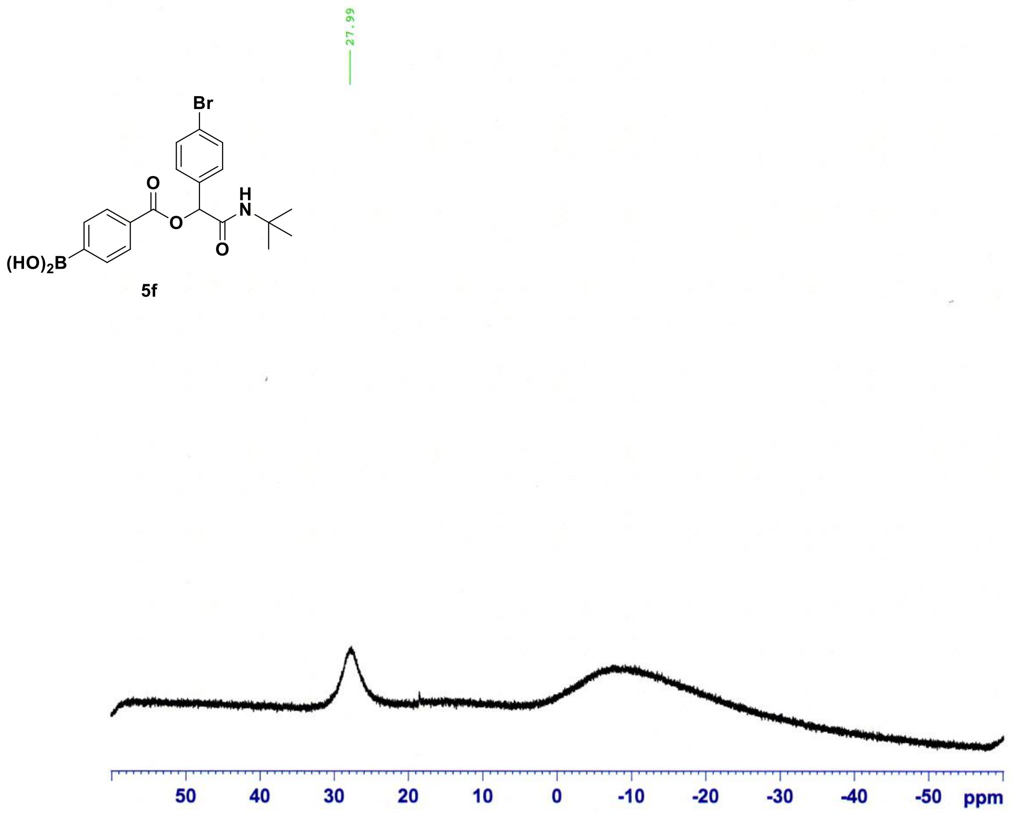

Figure S51. The ^{11}B NMR spectrum of **5f** in CD_3OD

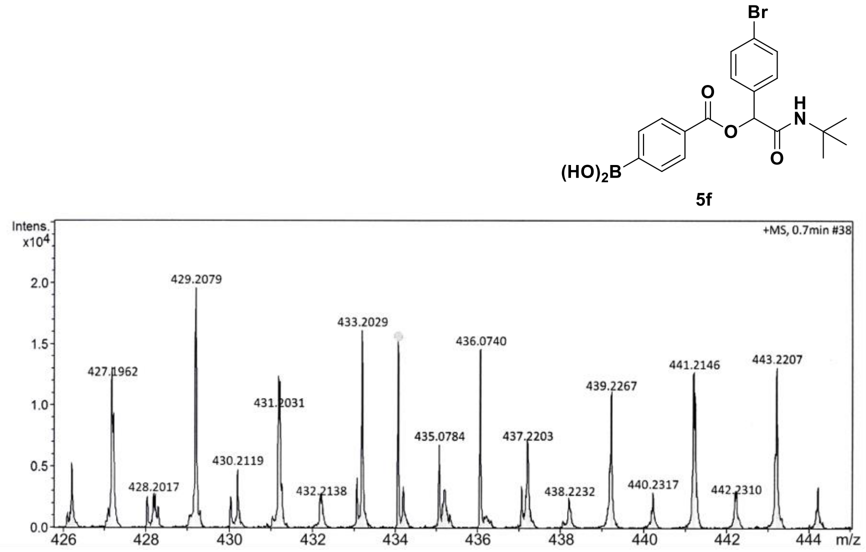

Figure S52. The HRMS (ESI^+) of spectrum of **5f**

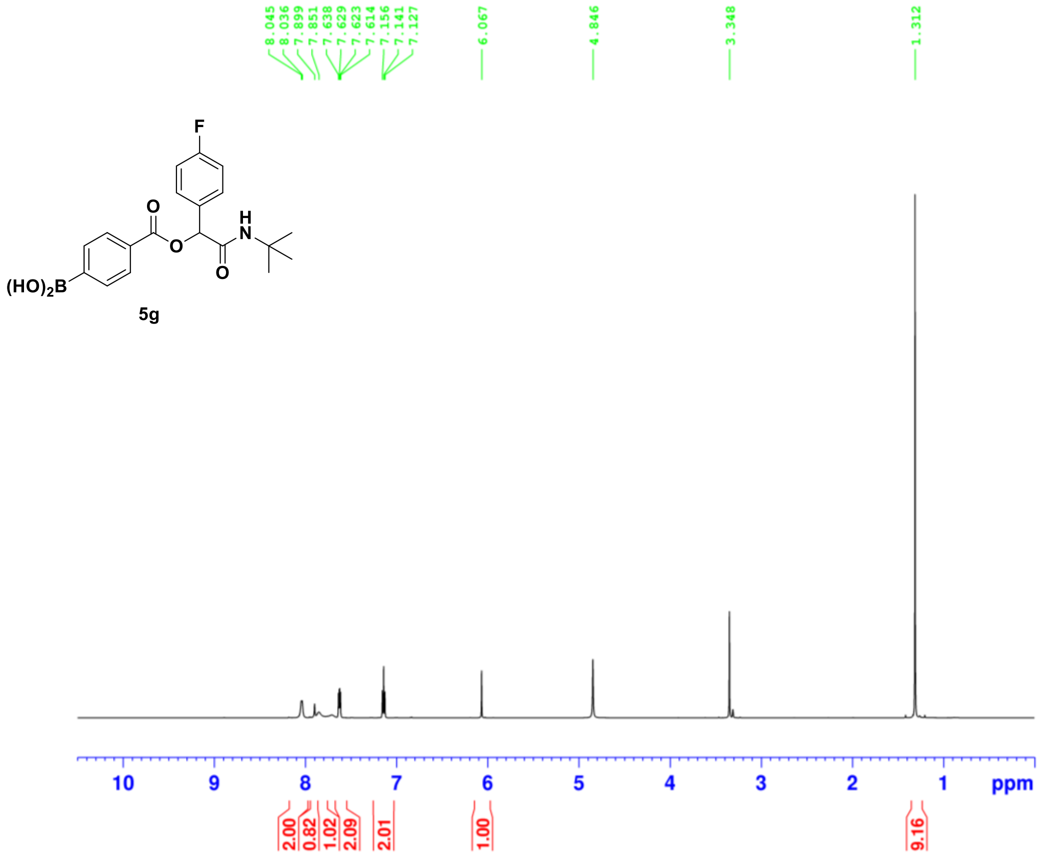

Figure S53. The ^{1}H NMR spectrum of **5g** in CD_3OD

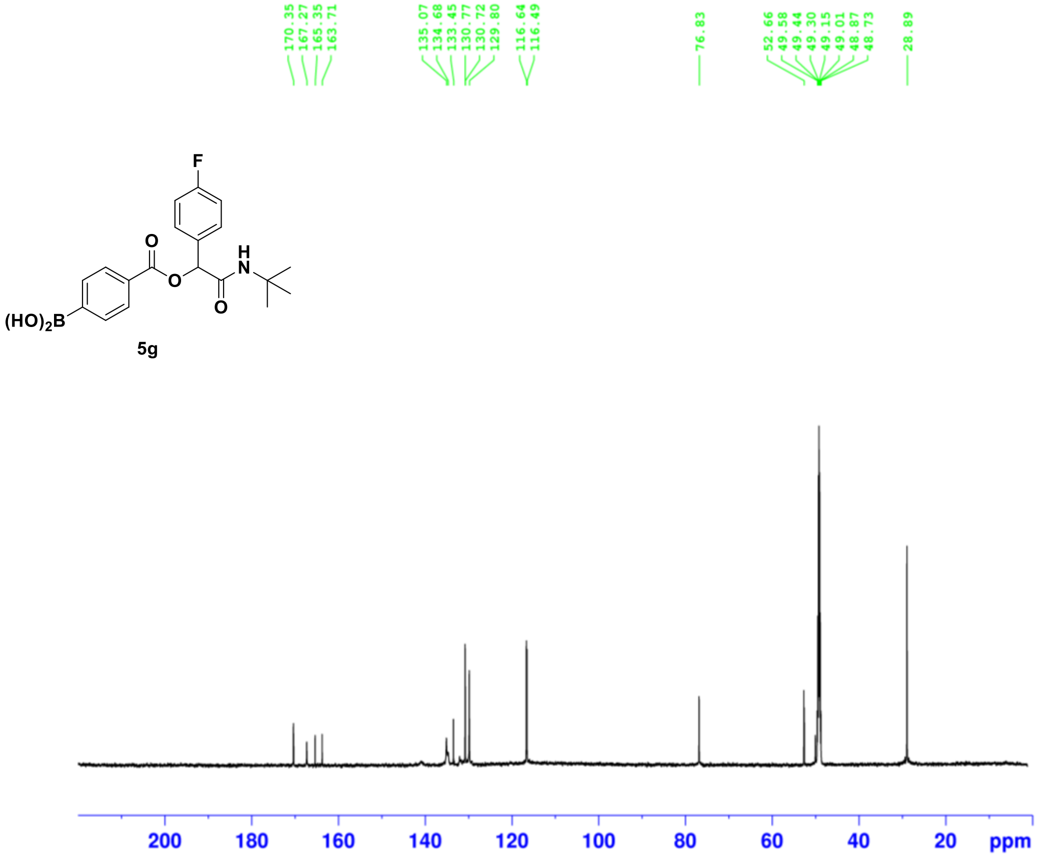

Figure S54. The ^{13}C NMR spectrum of **5g** in CD_3OD

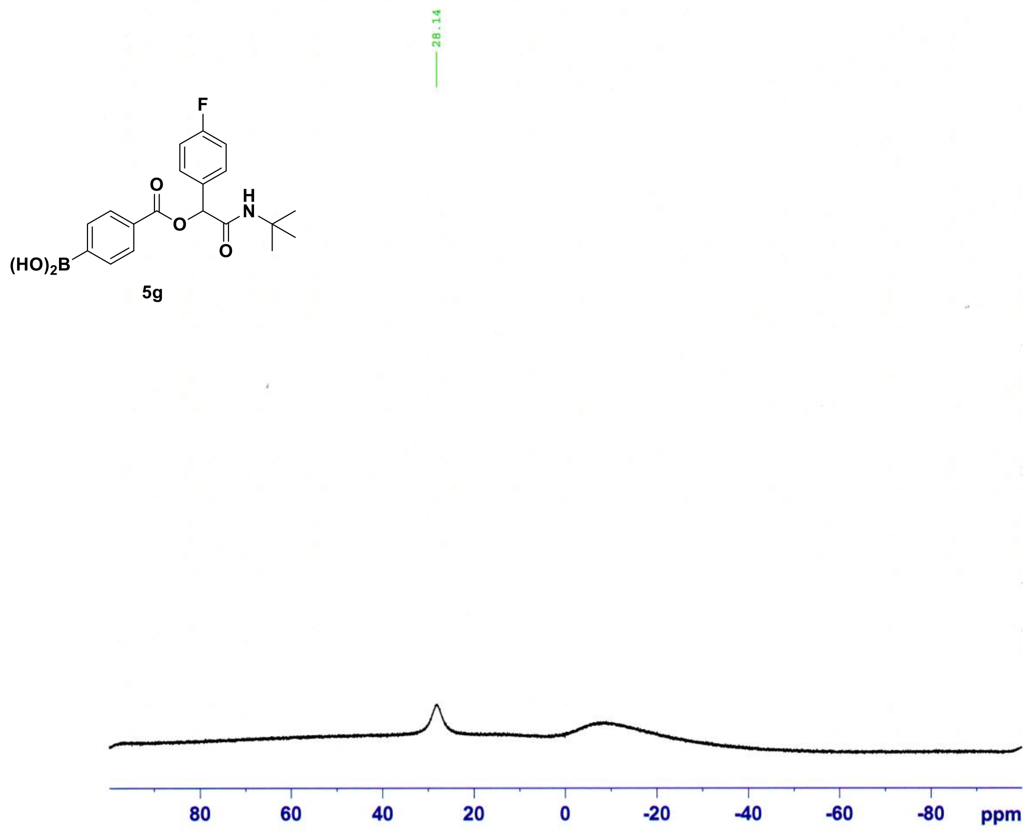

Figure S55. The ^{11}B NMR spectrum of **5g** in CD_3OD

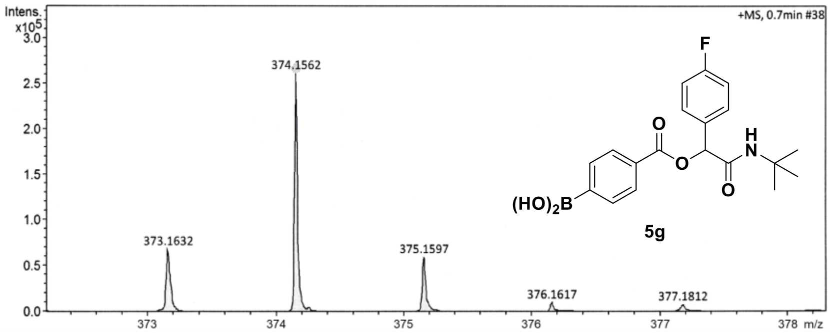

Figure S56. The HRMS (ESI+) of spectrum of **5g**

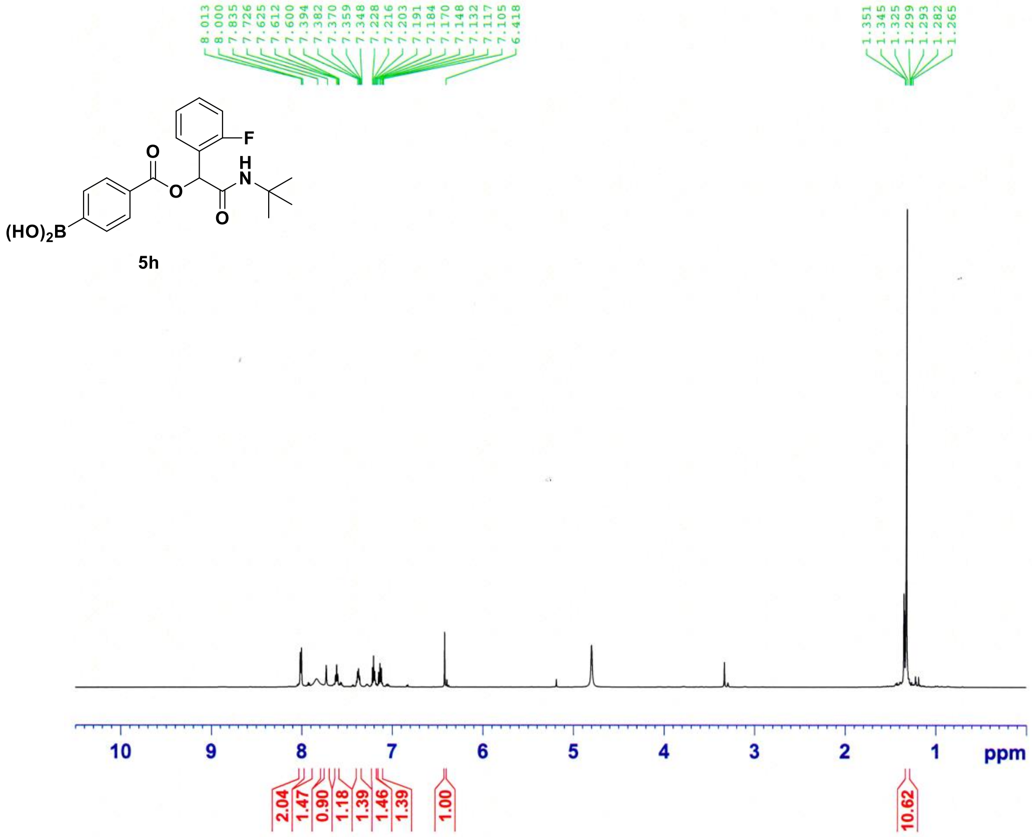

Figure S57. The ^{1}H NMR spectrum of **5h** in CD_3OD

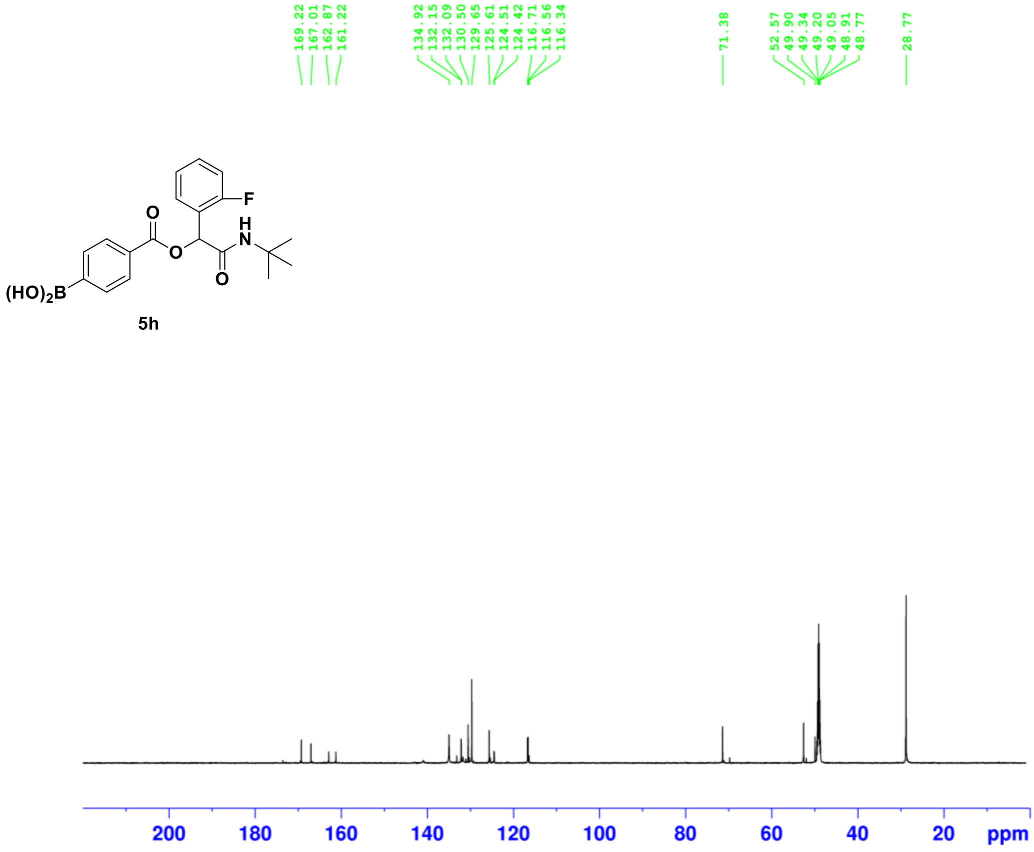

Figure S58. The ^{13}C NMR spectrum of **5h** in CD_3OD

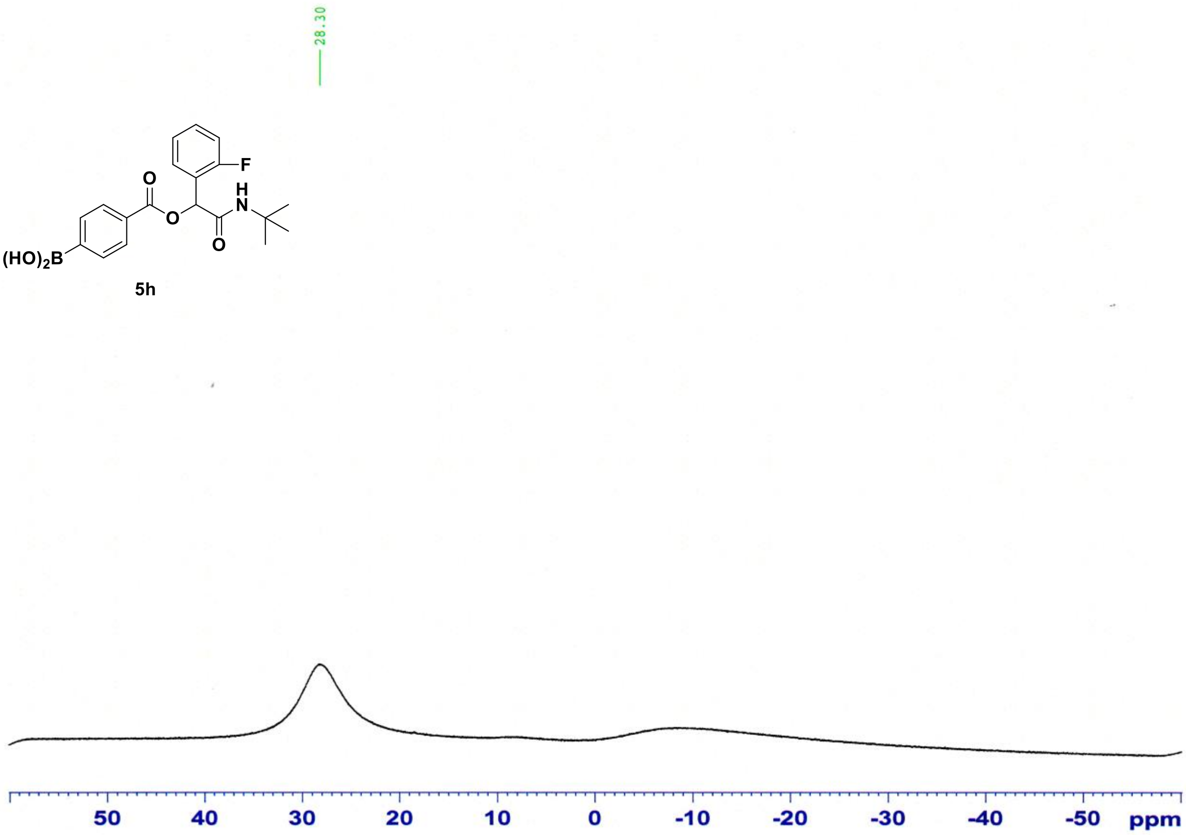

Figure S59. The ^{11}B NMR spectrum of **5h** in CD_3OD

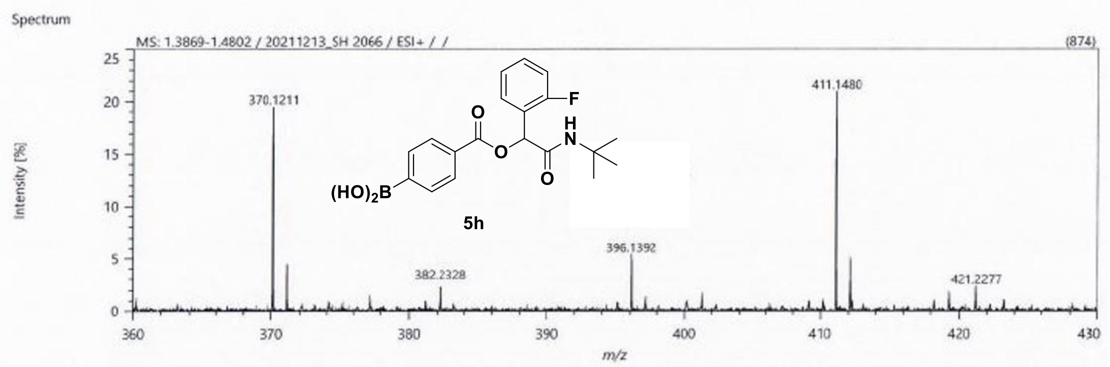

Figure S60. The HRMS (ESI$^+$) of spectrum of **5h**

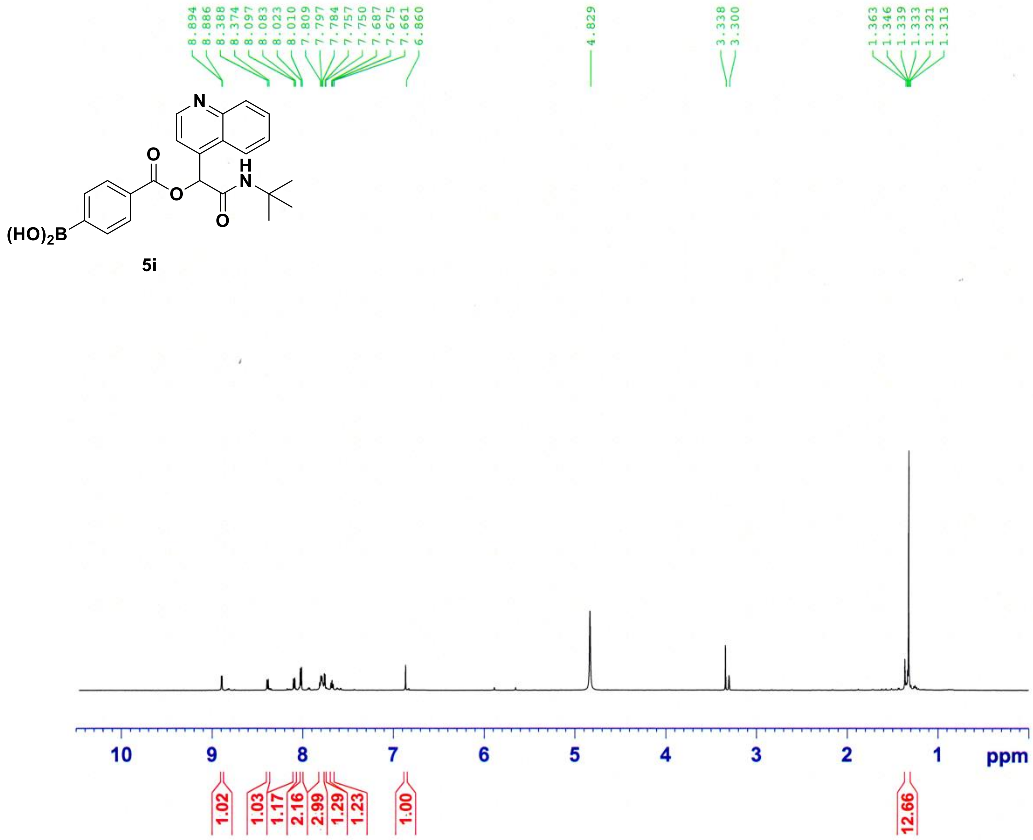

Figure S61. The ^{1}H NMR spectrum of **5i** in CD_3OD

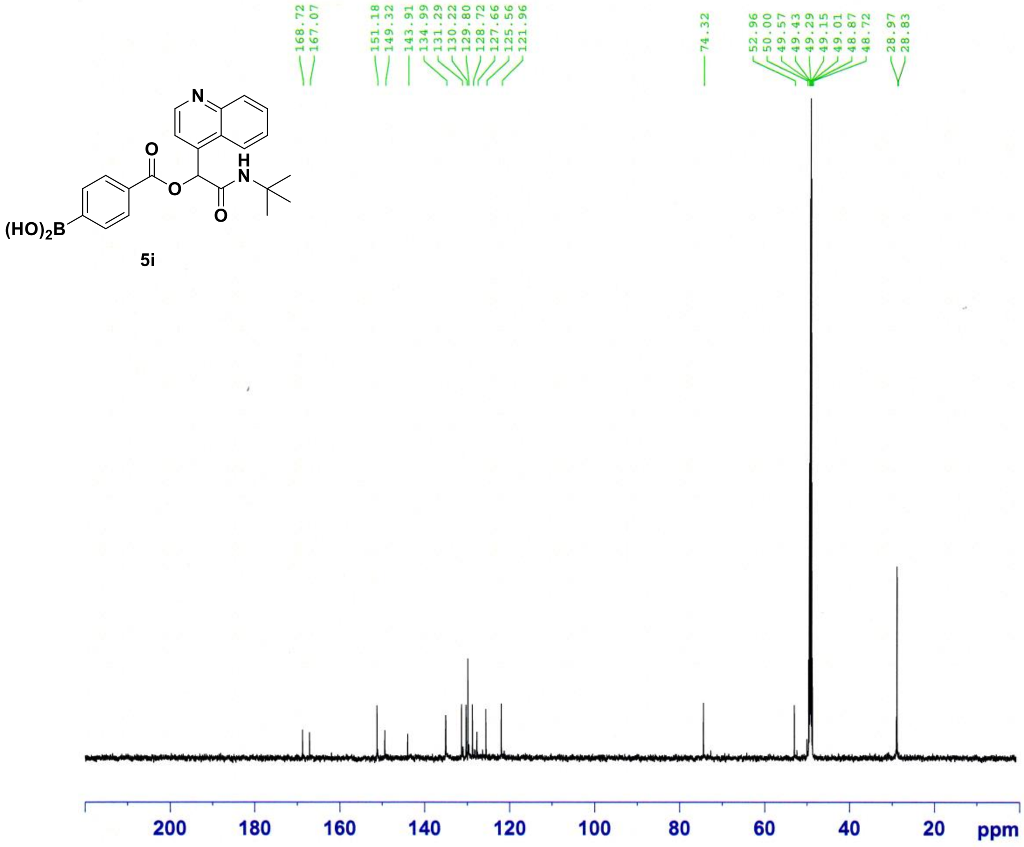

Figure S62. The ^{13}C NMR spectrum of **5i** in CD_3OD

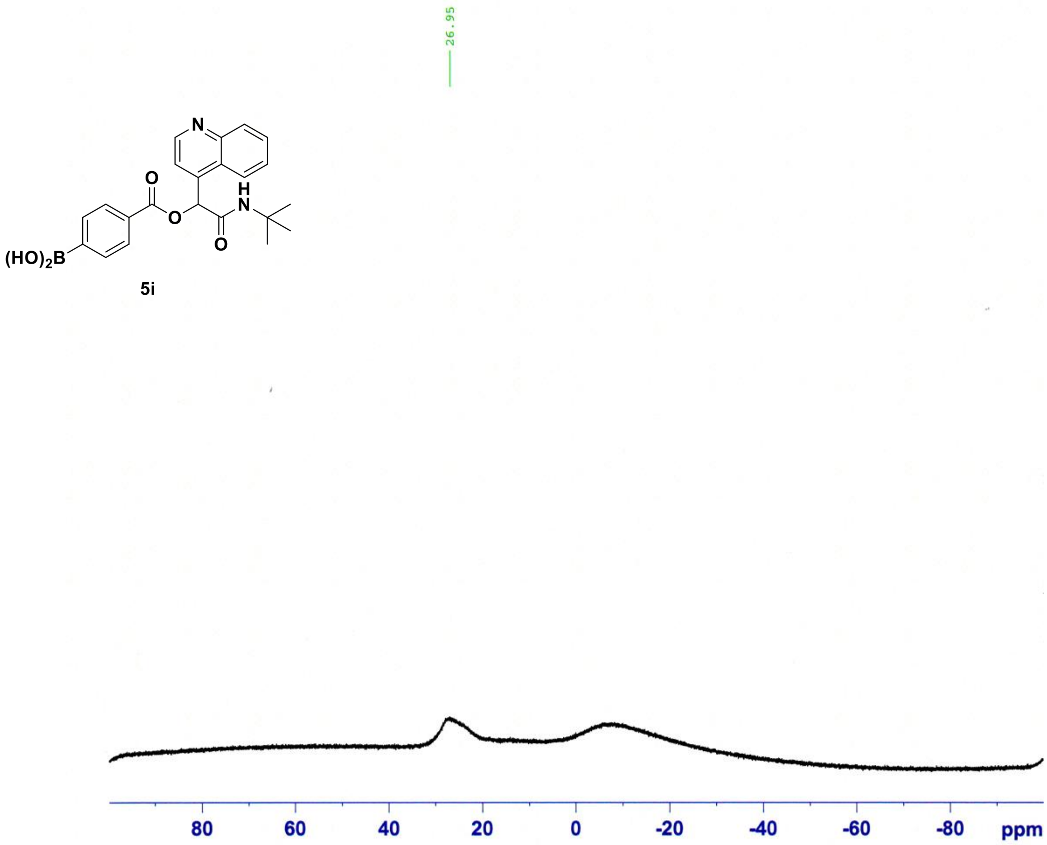

Figure S63. The ^{11}B NMR spectrum of **5i** in CD_3OD

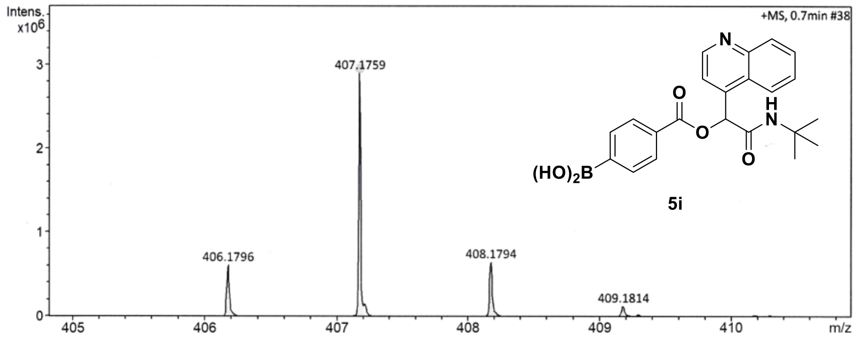

Figure S64. The HRMS (ESI^+) of spectrum of **5i**

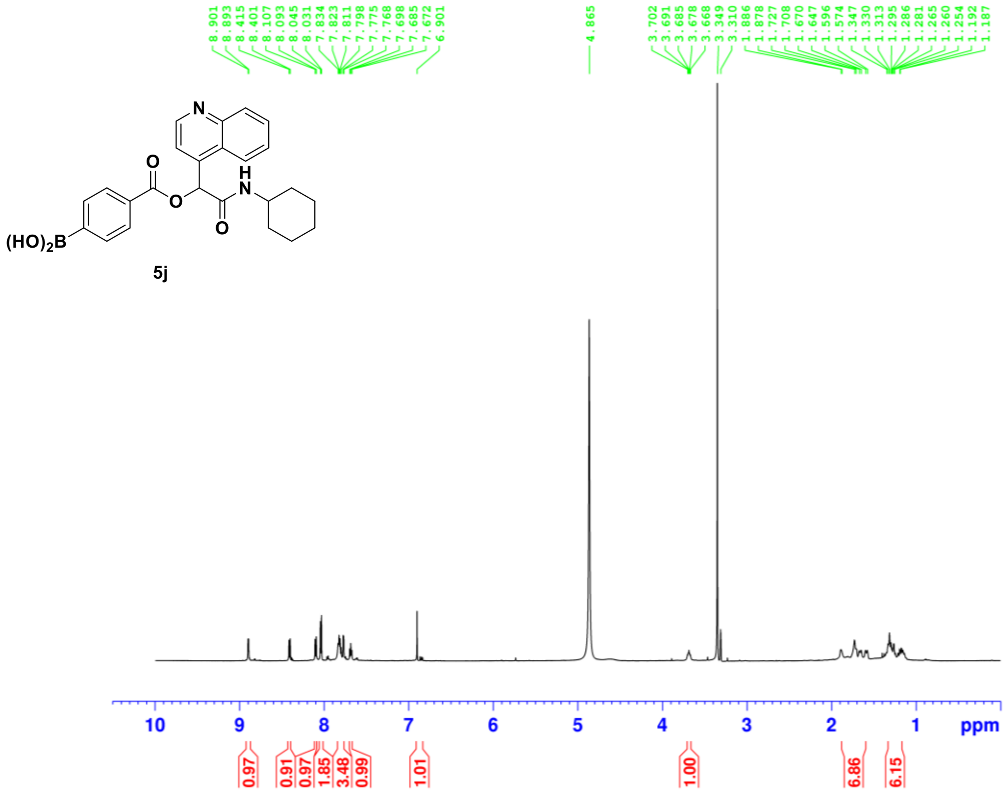

Figure S65. The ^{1}H NMR spectrum of **5j** in CD_3OD

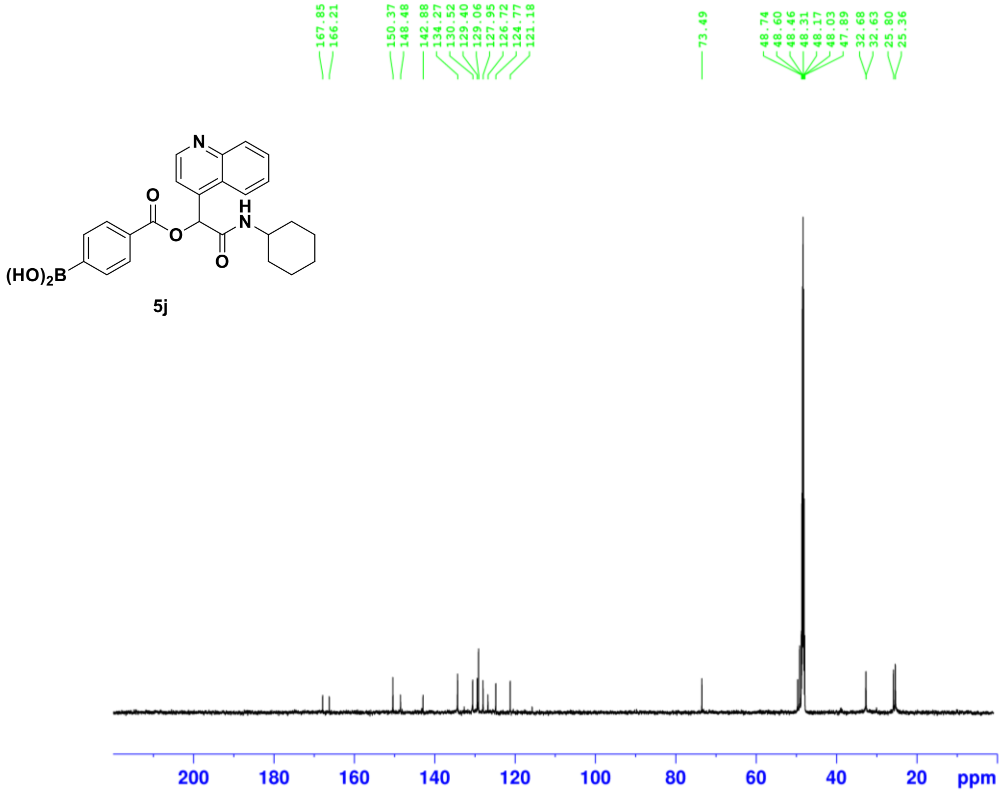

Figure S66. The ^{13}C NMR spectrum of **5j** in CD_3OD

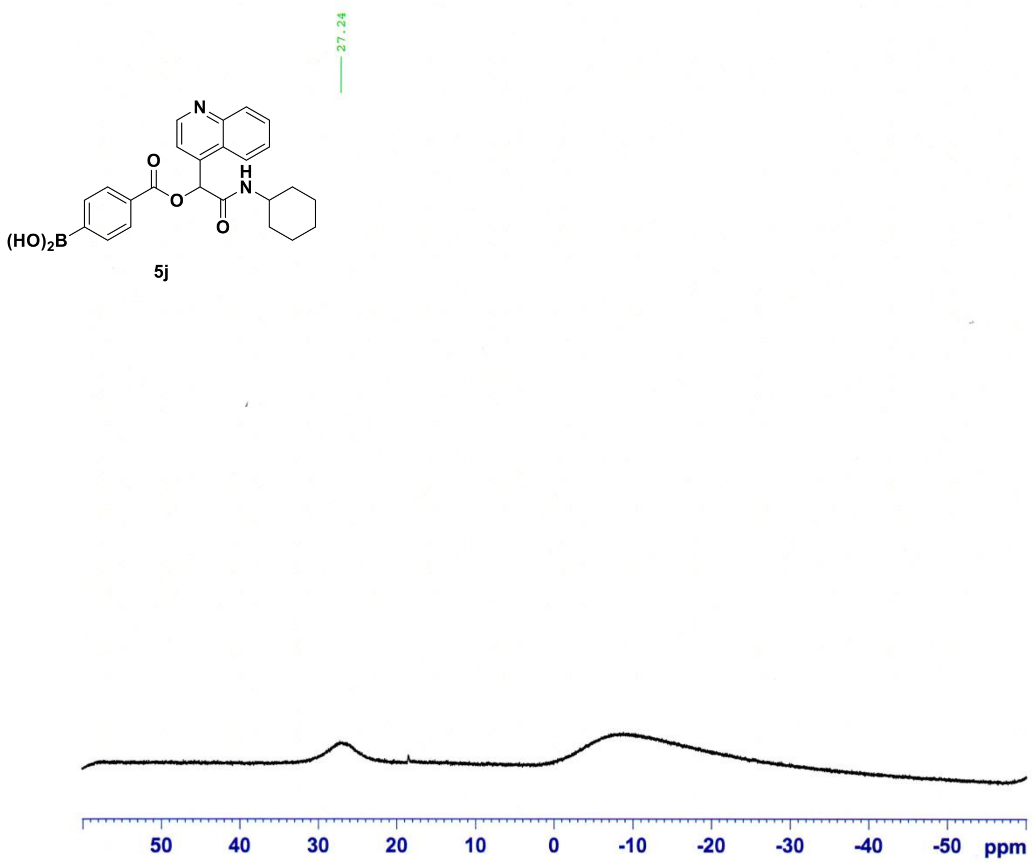

Figure S67. The ^{11}B NMR spectrum of **5j** in CD_3OD

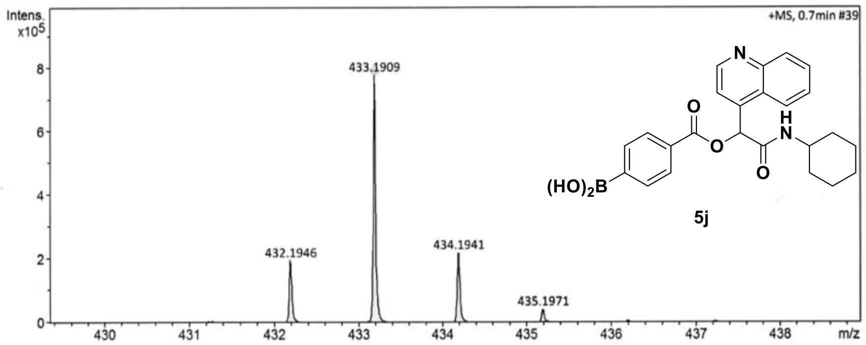

Figure S68. The HRMS (ESI+) of spectrum of **5j**

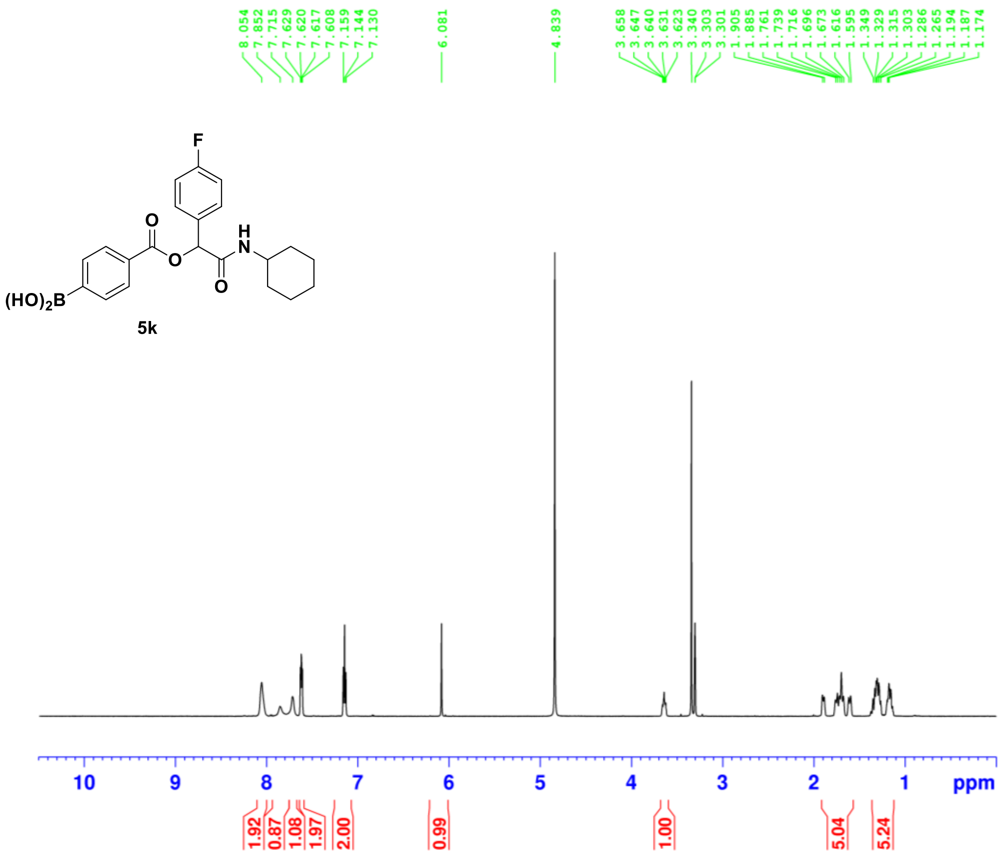

Figure S69. The ¹H NMR spectrum of **5k** in CD_3OD

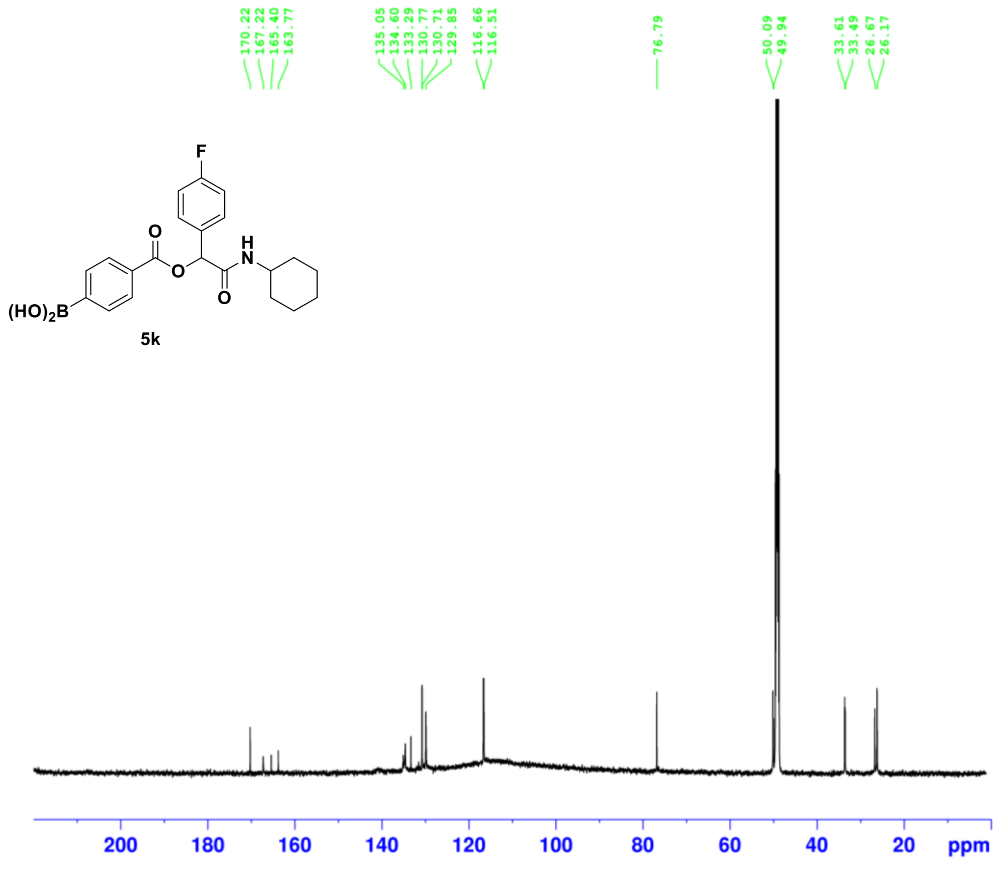

Figure S70. The ¹³C NMR spectrum of **5k** in CD_3OD

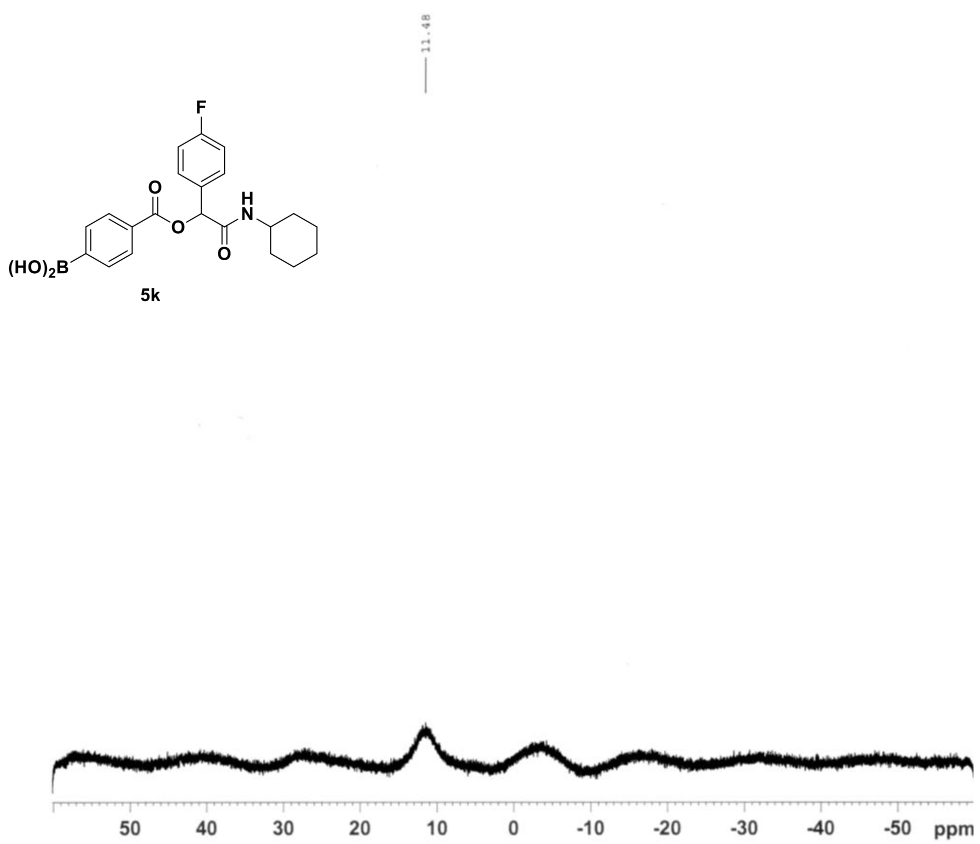

Figure S71. The ^{11}B NMR spectrum of **5k** in CD_3OD

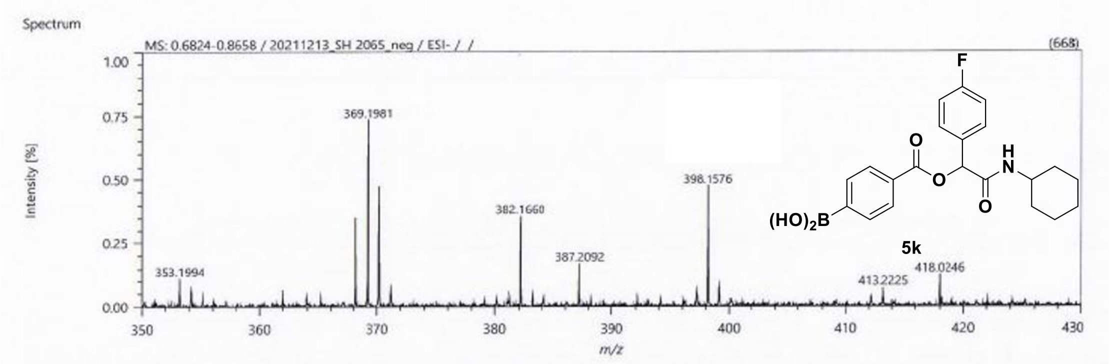

Figure S72. The HRMS (ESI$^-$) of spectrum of **5k**

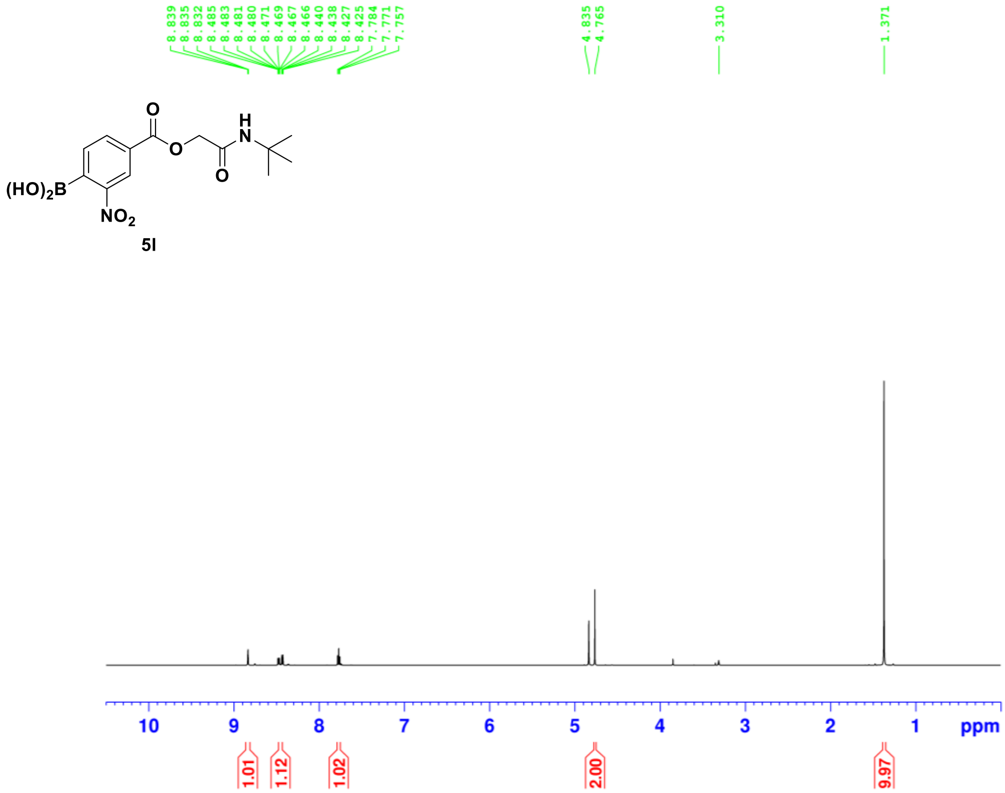

Figure S73. The ^{1}H NMR spectrum of **5l** in CD_3OD

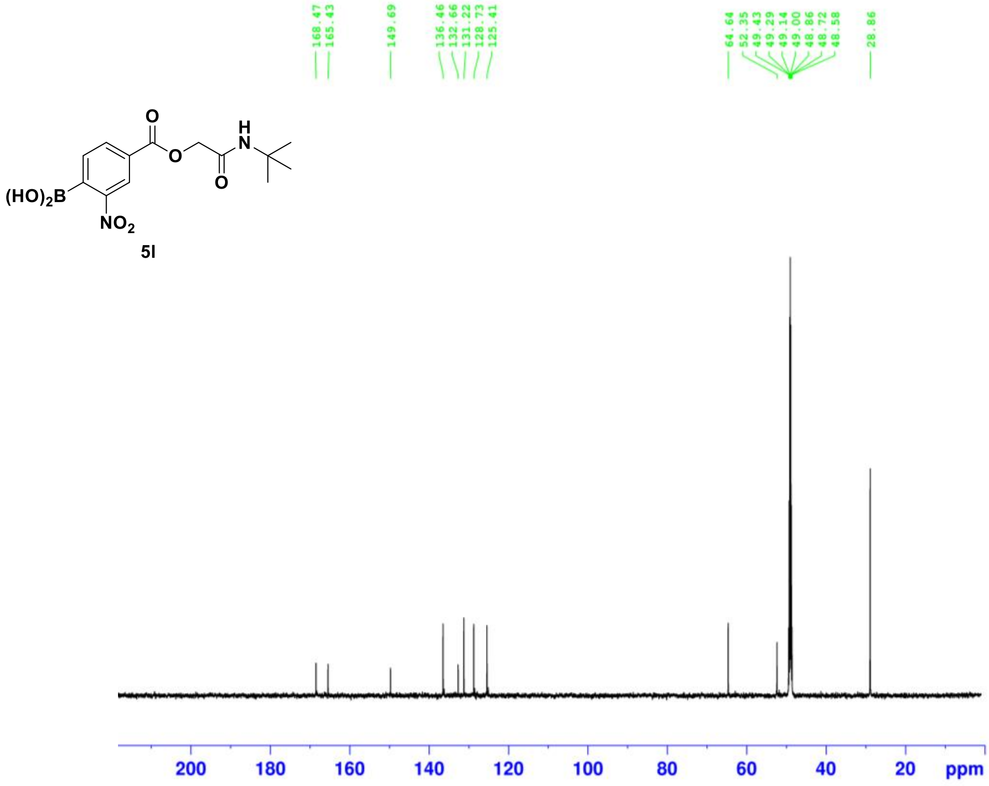

Figure S74. The ^{13}C NMR spectrum of **5l** in CD_3OD

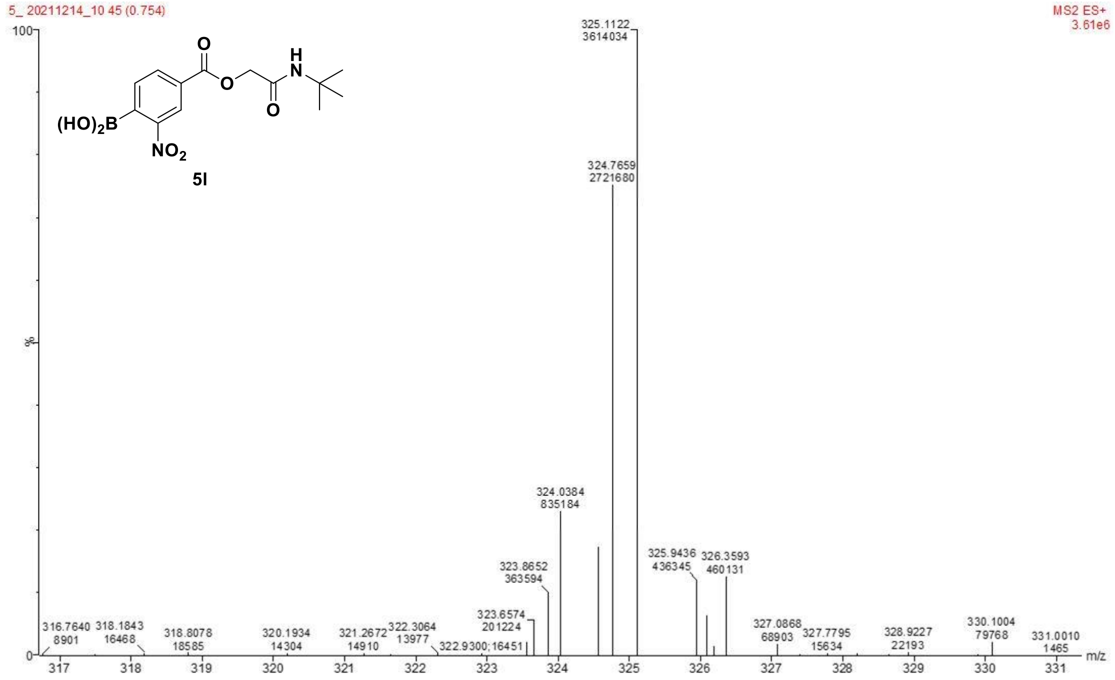

Figure S75. The HRMS (ESI+) of spectrum of **5l**

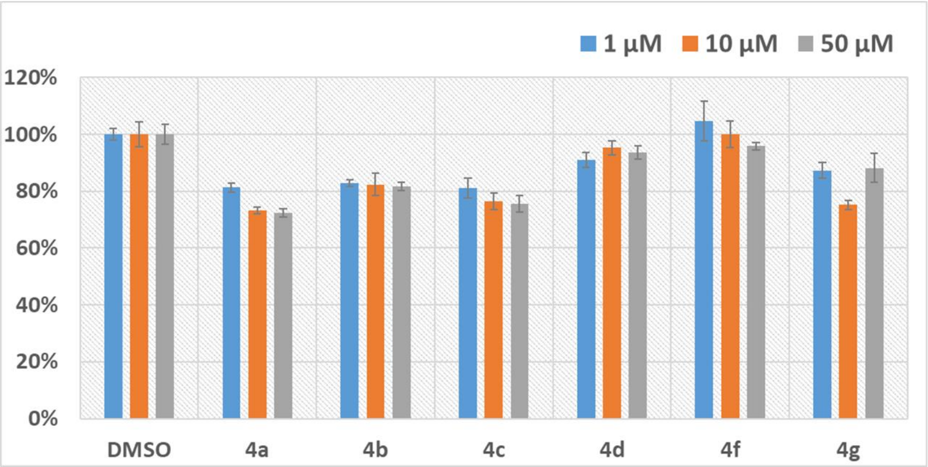

Figure S76. The cytotoxicity of the compounds **4a-4d**, **4f** and **4g** in HEK-293 cells.

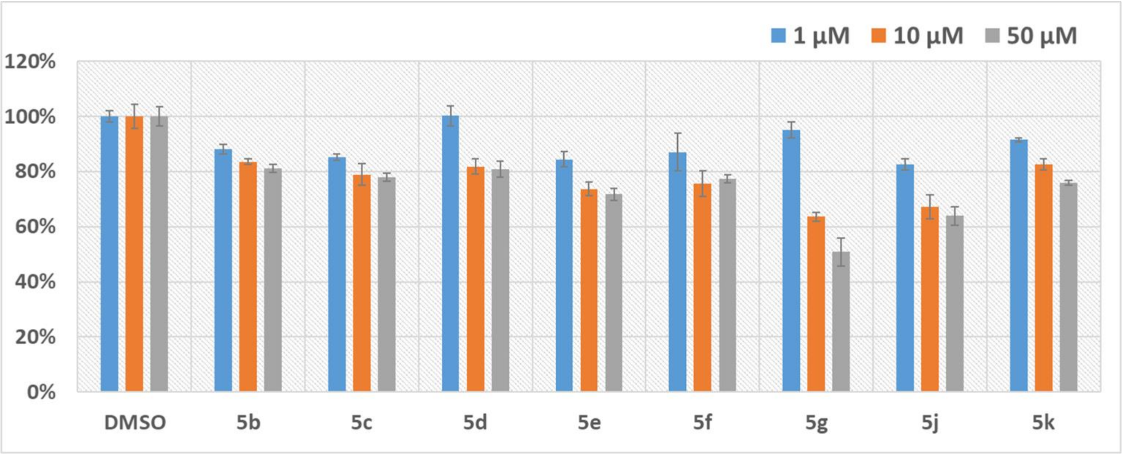

Figure S77. The cytotoxicity of the compounds **5b-5g**, **5j** and **5k** in HEK-293 cells.
